# Interleukin-10 regulates goblet cell numbers through Notch signaling in the developing zebrafish intestine

**DOI:** 10.1038/s41385-022-00546-3

**Published:** 2022-07-15

**Authors:** Rodrigo A. Morales, Soraya Rabahi, Oscar E. Diaz, Yazan Salloum, Bianca C. Kern, Mikaela Westling, Xinxin Luo, Sara M. Parigi, Gustavo Monasterio, Srustidhar Das, Pedro P. Hernández, Eduardo J. Villablanca

**Affiliations:** 1 Division of Immunology and Allergy, Department of Medicine Solna (MedS), Karolinska Institutet and University Hospital, 17176 Stockholm, Sweden; 2 Center for Molecular Medicine (CMM), 17176 Stockholm, Sweden; 3 Institut Curie, PSL Research University, INSERM U934, CNRS UMR3215, Development and Homeostasis of Mucosal Tissues Group, 75248 Paris, France

## Abstract

Cytokines are immunomodulatory proteins that orchestrate cellular networks in health and disease. Among these, interleukin (IL)-10 is critical for the establishment of intestinal homeostasis, as mutations in components of the IL-10 signaling pathway result in spontaneous colitis. Whether IL-10 plays other than immunomodulatory roles in the intestines is poorly understood. Here, we report that *il10*, *il10ra*, and *il10rb* are expressed in the zebrafish developing intestine as early as 3 days post fertilization. CRISPR/Cas9-generated *il10*-deficient zebrafish larvae showed an increased expression of pro-inflammatory genes and an increased number of intestinal goblet cells compared to WT larvae. Mechanistically, Il10 promotes Notch signaling in zebrafish intestinal epithelial cells, which in turn restricts goblet cell expansion. Using murine organoids, we showed that IL-10 modulates goblet cell frequencies in mammals, suggesting conservation across species. This study demonstrates a previously unappreciated IL-10-Notch axis regulating goblet cell homeostasis in the developing zebrafish intestine and may help explain the disease severity of IL-10 deficiency in the intestines of mammals.

## Introduction

The intestine is a semi-permeable and highly regenerative tissue responsible for nutrient and water absorption while keeping the host protected from environmental and microbiota challenges. The control of these functions is orchestrated by complex cellular crosstalk between intestinal epithelial cells and tissue-resident immune cells, among others. In this regard, cytokines, and particularly IL-10, have been considered key mediators in the maintenance of intestinal homeostasis and function.^1^ This has been demonstrated in mice defective in IL-10 signaling, which develop spontaneous colitis.^2^ In humans, mutations in the IL-10 signaling genes *IL10*, *IL10RA*, and *IL10RB* are strongly associated with a very early onset of inflammatory bowel diseases (VEO-IBD),^3,4^ which is characterized by clinical manifestations in children before 6 years of age, and a significant number of cases occurring in infants younger than 1 year.^5^ VEO-IBD has a strong genetic component compared to the 5–10% familial cases observed in adult inflammatory bowel diseases (IBD).^6^ In addition, VEO-IBD is phenotypically distinct from adult IBD and is characterized by extensive colitis, growth failure, and unresponsiveness to conventional therapies.^6^ Common clinical manifestations in children with VEO-IBD include bloody diarrhea and mucous stool,^7^ suggesting altered goblet cell activity.

Intestinal immune cells, including macrophages, B cells, and T cells, are the predominant IL-10 producers and responders. IL-10 signaling in intestinal macrophages is critical to maintaining intestinal homeostasis, as conditional deletion of IL-10Rα in CX3CR1^+^ macrophages is sufficient to trigger spontaneous colitis in mice.^8^ Interestingly, intestinal epithelial cells also express IL-10 receptors and are therefore equipped to mount a cellular response upon IL-10R stimulation^9–11^ In this regard, IL-10 deficiency in myeloid cells results in impaired regeneration of the epithelium following damage, whereas administration of recombinant IL-10 promotes intestinal epithelial cell proliferation in vivo.^12^ Furthermore, IL-10 promotes intestinal stem cell (ISC) proliferation in organoid cultures,^13^ suggesting an intrinsic role of IL-10 signaling in intestinal epithelial cells (IEC) differentiation. In line with this, specific IL-10R deficiency on IECs results in goblet cell hyperplasia in the colon of adult mice.^14^ Altogether, the combined evidence not only shows that IL-10 signaling can modulate IEC differentiation but also raises the possibility that early-stage intestinal development is impaired in individuals with mutations in the IL-10 signaling pathway, which may lead to the phenotypes observed in VEO-IBD.

The intestinal tract develops at early embryonic stages from the endodermal germinal layer. It is initially formed as a flat tube of epithelial and mesenchymal cells that proliferate and stratify in a synchronic fashion, which results in the morphogenesis of the villi and crypts. At the bottom of the crypt, intestinal stem cells are constantly self-renewing and giving rise to specialized epithelial cells with either absorptive or secretory functions. The main drivers of intestinal stem cell differentiation include the orchestrated activity of signaling pathways such as Wingless/Int-1 (Wnt), bone morphogenetic proteins (BMP), and Notch, which antagonize each other to promote or attenuate cell replication.^15,16^ Particularly, Notch signaling plays a critical function in stem cell maintenance and regulation of secretory cell differentiation across species.^17–19^ In mice, Paneth and goblet cells are expanded after Notch inhibition,^20^ and the lack of Notch target genes, such as *hairy/enhancer of split 1* (*Hes1*) show a skewed epithelial cell differentiation towards the secretory lineage.^21,22^ By contrast, continuous Notch activation by overexpression of the Notch intracellular domain (NICD) results in the expansion of the intestinal stem cell pool and impaired secretory-IEC differentiation.^23^ Expression of the Notch1 and Notch 2 receptors and their main target genes *Hes1*, *Hes5*, and *Hes6* have been detected in the dividing cells within the intestinal crypt.^24^ Interestingly, the Notch pathway regulates T cell function,^25^ including the production of IL-10 by Th1 cells.^26^ However, whether IL-10 signaling regulates tissue differentiation by controlling Notch or other stem cell-related pathways remains unknown.

Zebrafish have been successfully used to model key biological processes associated with intestinal disorders, as physiological and cellular features are highly conserved between fish and humans.^27^ Using zebrafish, we and others have addressed the impact of dietary compounds,^28^ environmental pollutants^29^ and IBD risk genes^30^ in intestinal homeostasis, highlighting zebrafish as a powerful tool to investigate the function of genetics in intestinal development. The zebrafish ortholog for the human *IL10* gene^31^ shows a conserved immunomodulatory function.^32,33^ Here, we took advantage of zebrafish to investigate the effect of IL-10 signaling in the developing intestine. Using CRISPR/Cas9 we generated Il10 mutants, which combined with histological and immunological stainings, transcriptional analysis and fluorescent transgenic reporters enabled us to investigate the intestinal cell composition of IL-10-deficient larvae. We found that IL-10 deficiency resulted in an increased number of goblet cells, which was independent of the microbiota. Mechanistically, the increase in goblet cells was associated with a decreased activity of intestinal Notch signaling, and ectopic Notch signaling activation restored alcian blue^+^ goblet cell numbers in IL-10-deficient larvae. Moreover, IL-10 treatment in mouse small intestine (SI) organoids led to decreased frequencies of goblet cells and increased Notch activity, which suggests an evolutionarily conserved IL-10-Notch axis that regulates intestinal goblet cell differentiation across vertebrates.

## Results

### Zebrafish *il10* is expressed during early intestinal development

**Fig. 1:**
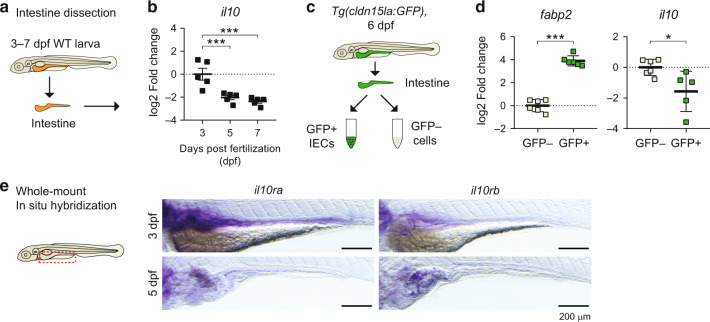
Early expression of *il10* during intestinal development in zebrafish larvae. **a** Diagram showing intestine extractions of wild-type (WT) larvae, from 3 to 7 days post fertilization (dpf). **b** Expression of *il10* transcripts measured by qRT-PCR. Each dot represents independent experiments with a pool of 20 intestines used for RNA extraction (*N* = 5). **c** Sorting strategy for the isolation of intestinal epithelial cells. Intestines from *Tg(cldn15la:GFP)* larvae were extracted from the body, disaggregated to cell suspensions, and FACS sorted based on the expression of GFP. GFP^+^ and GFP^-^ cell collections were subsequently used for RNA transcriptomic analyses. **d** Transcriptomic expression analyses from sorted GFP^+^ and GFP^-^ cells by qRT-PCR. Each dot represents an individual sorting experiment with around 30,000 cells collected (*N* = 6). **e** Whole-mount in situ hybridization for the genes *il10ra* and *il10rb* in 3dpf and 5dpf zebrafish larvae. Representative pictures of the stainings at each developmental stage are shown. Scale bar = 200 µm. One-way ANOVA with Fisher’s LSD multiple comparisons test was performed in **b**, while two-tailed *t*-tests were performed in **d** (*p < 0.05; ***p < 0.001).

The development of the larval zebrafish intestine, after the formation of the tube and lumen between 30 and 52 hours post fertilization (hpf), can be divided into an early stage of high proliferation and polarization of the intestinal epithelium (52–74hpf), followed by a later stage of tissue compartmentalization and the emergence of differentiated intestinal cell types (74–120 hpf).^34,35^ To gain insights into the developmental stage in which Il10 might control zebrafish intestinal organogenesis, we analyzed *il10* transcript levels in zebrafish larvae starting at 72 hpf or 3 days post fertilization (dpf) until the stage in which a functional intestine is appreciated (5dpf and 7dpf). Using qRT-PCR to analyze *il10* transcript levels from dissected zebrafish larval intestines (Fig. 1a), we found higher transcript levels of *il10* in 3dpf intestines, compared to 5dpf and 7dpf (Fig. 1b). Dissected intestines contain intestinal epithelial cells (IECs) among other intestine-associated cells that interact with IECs, therefore we sought to determine which group of cells was responsible for *il10* expression. We then FACS-sorted epithelial cells versus the rest by using the zebrafish reporter line *Tg(cldn15la:GFP)* (Fig. 1c), in which the *cldn15la* promoter drives GFP expression exclusively in intestinal epithelial cells.^36^ qPCR analysis from sorted cells showed that *il10* expression was not detectable in GFP^+^ cells but in GFP^-^ intestine-associated cells (Fig. 1d). To gain insights into a potential active IL-10 signaling in intestinal cells, we analyzed the expression of the orthologs for the receptors of *il10*, defined as *il10ra* and *il10rb* in the zebrafish database ZFIN. Whole-mount in situ hybridizations (WISH) showed that both *il10ra* and *il10rb* genes were expressed in the zebrafish developing intestine at 3dpf, while *il10rb* was predominantly expressed at 5dpf (Figs. 1e and S1). Altogether, these results suggest that *il10* signaling may act in zebrafish intestinal cells during early developmental stages.

### Altered immune and intestine-associated profiles in *il10* mutant larvae

**Fig. 2:**
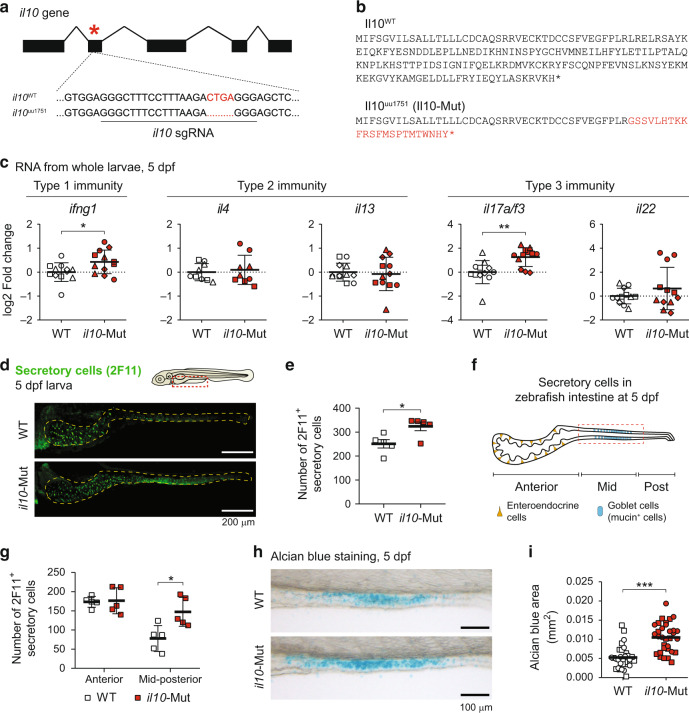
Increased pro-inflammatory expression and alcian blue^+^ goblet cells in *il10* mutant zebrafish larvae. **a** Schematic for the mutation generated in the zebrafish *il10* gene (*il10*^uu1751^, -4bp) by CRISPR/Cas9. **b** Predicted protein sequences for Il10 in WT and *il10*-Mut individuals, according to the DNA sequences obtained. **c** Whole-body expression levels of type 1, type 2, and type 3 cytokines in WT (white symbols) and *il10*-Mut larvae (red symbols) by qRT-PCR. Each dot represents a pool of 5–10 larvae collected in 3–4 independent experiments. **d** Whole-mount immunofluorescence staining in 5dpf WT and *il10*-Mut larvae using the pan-secretory marker antibody 2F11. Scale bar = 100 µm. **e** Quantification of 2F11^+^ cells in the intestines of WT and *il10*-Mut larvae. Each dot represents individual larvae collected in 2 independent experiments. **f** Diagram for the larval zebrafish intestinal tract showing the location of Goblet cells in the mid-intestinal region. **g** Stratification of the number of 2F11^+^ cells in the anterior or mid-posterior intestines of WT and *il10*-Mut larvae (*N* = 5). **h** Alcian blue (ab) staining on 5dpf WT and *il10*-Mut larvae. **i** Automatic quantifications of the ab-stained area in the mid intestines of WT and *il10*-Mut larvae. Each dot corresponds to an individual larva collected in two independent experiments. Different symbol shapes represent independent experiments in **c** and **i**. Two-tailed student *t* tests were used for the comparisons in **c**, **e**, **g** and **i**. (*p < 0.05; **p < 0.01; ***p < 0.001).

Next, we aimed to determine whether *il10* can modulate the development of the larval zebrafish intestine by using loss-of-function genetic approaches. Using CRISPR/Cas9,^37^ we generated a 4 bp deletion in the exon 2 of the zebrafish *il10* gene (*il10*^uu1751^) (Fig. 2a), predicted to generate a premature stop codon (Fig. 2b). *il10*^uu1751/uu1751^ mutant fish (hereinafter referred to as *il10*-Mut) did not show any apparent morphological or developmental defect while in homozygosis in larva (Fig. S2a), reached adulthood and were fertile (Fig. S3a, b). Analysis of selected type 1, 2, and 3 cytokines by qPCR from the whole larvae at 5dpf revealed that type 2 cytokines (*il4* and *il13*) and the type 3 cytokine *il22* were comparable whereas *ifng1* (type 1) and *il17a/f3* (type 3) were significantly elevated in *il10*-Mut larvae compared to wild-type (WT, *il10*^wt/wt^) larvae (Figs. 2c and S2b). Thus, Il10 deficiency results in altered pro-inflammatory cytokine levels in the whole larvae. We then sought to investigate whether Il10 deficiency affects the development of the intestinal tract. Absorptive and secretory cells, namely enteroendocrine and goblet cells, can be distinguished by 5dpf in zebrafish larvae.^18^ To analyze enteroendocrine and goblet cells, we performed whole-mount immunofluorescence using the pan-secretory marker antibody 2F11.^18^ By 5dpf, the *il10*-Mut larvae showed a higher number of secretory cells in the intestine compared to WT (Fig. 2d, e). Secretory cells in the zebrafish larvae are differentially distributed along the anterior-distal intestine,^34^ with enteroendocrine cells being concentrated in the anterior intestine and mucin-producing goblet cells in the mid intestine (Fig. 2f). Therefore, we further stratified 2F11^+^ cells based on location. We observed that the number of 2F11^+^ secretory cells in the mid-posterior, but not in the anterior segment, was significantly increased in *il10*-Mut compared to WT (Fig. 2g). The increase in secretory cells in the mid-posterior segment prompted us to hypothesize that goblet cells are increased in *il10*-Mut compared to WT larvae. In line with our hypothesis, whole-mount alcian blue (ab) staining labeling mucin production as an indicator of ab^+^ goblet cell abundance in the mid intestine, displayed a higher staining area in 5dpf *il10*-Mut larvae compared to WT (Fig. 2h–i). Area measurements were complemented with ab intensity and stained length (Fig. S2c), showing an overall increase of the ab^+^ signal detected in *il10*-Mut larvae. Likewise, whole-mount staining with fluorescently labeled wheat germ agglutinin (WGA), which binds to the N-acetylglucosamine present in the mucus produced by goblet cells^34^ showed that the number of WGA^+^ goblet cells in the mid intestine was increased in 5dpf *il10*-Mut larvae, compared to WT (Fig. S2d, e), further confirming that *il10*-deficiency results in goblet cell hyperplasia within the developing zebrafish embryo. To analyze if absorptive intestinal populations were affected in *il10*-Mut larvae, we used neutral red to stain a subset of mid intestine absorptive enterocytes with acidified lysosomes, namely lysosome-rich enterocytes (LREs).^38–40^ Area, intensity and length analysis from neutral red-stained enterocytes in 5dpf larvae did not show differences between WT and *il10*-Mut (Fig. S2f, g), suggesting no impairments in the differentiation of absorptive intestinal lineages. Further, to determine whether the increased number of goblet cells in *il10*-Mut zebrafish was age-dependent, we analyzed different intestinal regions from >1-year-old WT and *il10*-Mut zebrafish (Fig. S3c). Ab staining on paraffin-embedded intestinal sections showed a higher number of goblet cells per villus area in *il10*-Mut larvae, compared to WT (Fig. S3d, e). Altogether, our results point towards a disbalance in both pro-inflammatory cytokine expression and the abundance of goblet cells in *il10*-deficient zebrafish.

### The abundance of intestinal goblet cells depends on *il10*

**Fig. 3:**
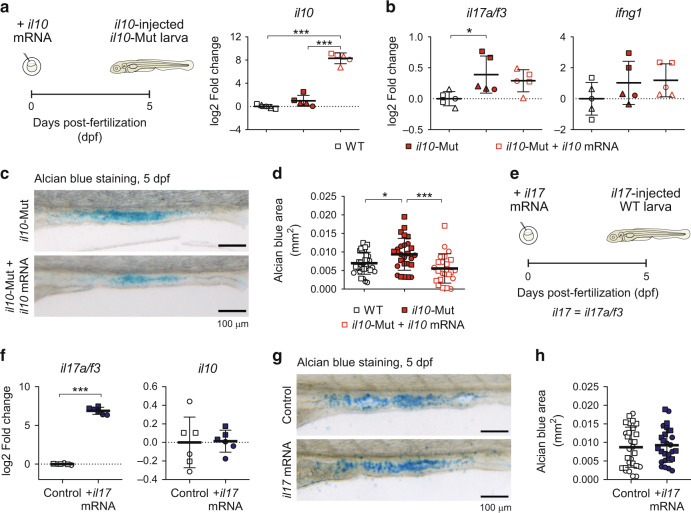
Altered alcian blue^+^ goblet cell homeostasis in *il10* mutant larvae is rescued after *il10* mRNA administration. **a** Experimental strategy for *il10* rescue experiments and expression levels of *il10* mRNA at 5dpf. **b** Whole-body expression levels of *il17a/f3* and *ifng1* by qRT-PCR. Each dot represents a pool of 5–10 larvae collected in three independent experiments. **c** Alcian blue stainings on 5dpf WT, *il10*-Mut larva controls and injected with *il10* mRNA. Scale bar = 100 µm. **d** Quantification for the ab-stained area in the *il10* mRNA-injected *il10*-KO larvae, compared to control *il10*-KO and WT larvae. *N* = 2 independent experiments. **e** Experimental strategy for the injection and analysis of *il17* (*il17a/f3*) mRNA-injected WT zebrafish embryos. **f** Whole-body expression levels of *il17a/f3* and *il10* by qPCR. Dots represent pools of 5–10 larvae collected in 2 independent experiments. **g** Alcian blue stainings on 5dpf control and *il17*-injected WT larvae. Scale bar = 100 µm. **h** Quantification of the ab-stained area of control and *il17*-injected larvae. *N* = 2 independent experiments. Each independent experiment is shown with a different symbol shape in **a**, **b**, **d**, **f** and **h**. One-way ANOVA was used for statistical analysis in **a**, **b** and **d**, whereas two-tailed student t-test were used in **f** and **h** (*p < 0.05; ***p < 0.001).

To determine whether the phenotypes observed in the *il10*-Mut were dependent on Il10 signaling rather than off-target effects we performed rescue experiments using an in vitro-generated version of the zebrafish *il10* mRNA. Injection of the full *il10* mRNA at 1-cell stage resulted in a significant increase of *il10* transcripts in the whole 5dpf larvae (Fig. 3a). *il10* mRNA-injected *il10*-Mut 5dpf larvae showed a non-significant reduction of *il17a/f3* transcript levels compared to *il10*-Mut, whereas *ifng1* levels were comparable (Fig. 3b). In addition, the average ab-stained area was reduced in the intestines of *il10* mRNA-injected *il10*-Mut larvae compared to *il10*-Mut, reaching comparable levels to those of WT larvae (Fig. 3c, d). A recent publication showed that IL-17 signaling on intestinal epithelial cells promotes goblet cell differentiation.^41^ To assess the role of Il17 signaling on the increased goblet cell numbers observed in *il10*-Mut larvae, we analyzed the abundance of intestinal goblet cells in WT larvae injected with an in vitro-generated *il17* (*il17a/f3*) mRNA at 1-cell stage (Fig. 3e). While *il17a/f3* transcripts were high in 5dpf *il17*-injected larvae (Fig. 3f), ab analysis did not show differences in the ab-stained area between *il17a/f3* mRNA-injected and control larvae (Fig. 3g, h), suggesting that the abundance of goblet cells does not depend on Il17 signaling in zebrafish larvae. To further confirm our findings, we measured the ab-stained area in a second CRISPR/Cas9-generated mutant line for the *il10* gene, this time containing a 17 bp insertion replacing 3 bp (+14 bp total insertion) in exon 3 (*il10*^uu1762^, referred to as *il10*-Mut2), which is predicted to generate a premature stop codon (Fig. S4a, b). Similar to the *il10*-Mut, we observed increased staining for ab in 5dpf *il10*-Mut2 larvae compared to WT (Fig. S4c, d). Altogether, these results indicate that *il10* modulates ab^+^ goblet cell differentiation, but not cytokine expression, in zebrafish larvae.

### Increased goblet cells in *il10* mutant larvae are microbiota-independent

**Fig. 4:**
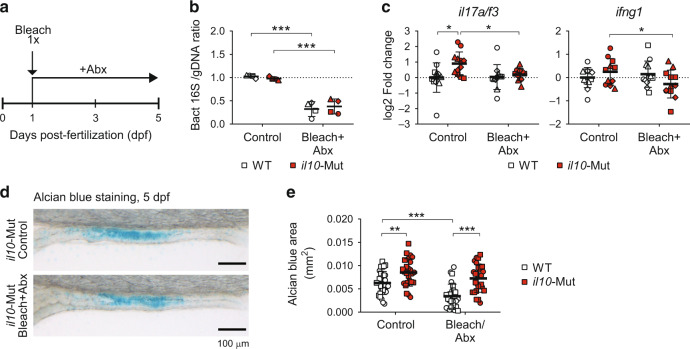
Increased alcian blue^+^ goblet cells in *il10* mutant larvae are independent of the microbiota. **a** Diagram showing bleach (sodium hypochlorite 0.004%) plus antibiotic (Abx) treatment in zebrafish embryos/larvae (Abx: Ampicillin 100 µg/mL, Kanamycin 5 µg/mL). **b** Quantification of bacterial 16S DNA levels over zebrafish genomic DNA measured by PCR. Each dot corresponds to a pool of 2 larvae from 4 independent experiments. **c** Transcriptomic expression analysis of cytokines from treated larvae by qRT-PCR. Each dot represents a pool of 8–10 larvae collected from 4 independent experiments (**d**, **e**) Alcian blue staining and analysis on 5dpf WT and *il10*-Mut larvae treated with Bleach+Abx and controls (*N* = 2 independent experiments). Scale bar = 100 µm. Independent experiments are shown with different symbol shapes in **b**, **c** and **e**. Two-way ANOVAs with Fisher’s LSD multiple comparisons tests were performed in **b**, **c** and **e** (*p < 0.05; **p < 0.01; ***p < 0.001).

Previous findings suggest that goblet cell differentiation is dependent on the microbiota.^42^ To investigate a potential role of the microbiota in the phenotypes observed in *il10*-Mut larvae, we established a protocol to reduce bacterial loads in zebrafish embryos (Fig. 4a). Briefly, 24–28 hpf embryos were treated with E3 water containing 0.004% sodium hypochlorite (bleach) for 5 min, and then they were incubated in the presence of an antibiotic cocktail (Abx, Ampicillin and Kanamycin) from 1dpf to 5dpf. We confirmed a reduction of total bacterial loads after bleach+Abx treatments in larvae by comparing bacterial 16S versus targeted zebrafish genomic DNA amplification levels by PCR (Fig. 4b). Bleach + Abx-treated 5dpf *il10*-Mut larvae showed a reduced expression of both *il17a/f3* and *ifng1*, compared to non-treated siblings (Fig. 4c), suggesting that cytokine modulation is microbiota-dependent. On the other hand, ab levels remained enhanced in *il10*-Mut compared to WT larvae, regardless of the reduced bacterial load (Fig. 4d, e). These results indicate that the increased ab^+^ goblet cells observed in *il10*-Mut larvae are independent of the bacterial microbiota.

### Impaired Notch signaling in the intestines of *il10* mutant larvae

**Fig. 5:**
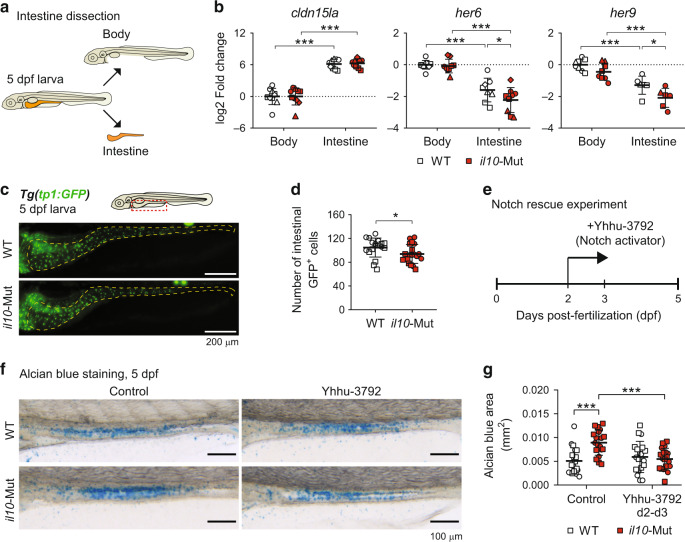
Decreased Notch signaling activity in the intestines of *il10* mutant larvae. **a** Schematics showing the body-intestine tissue collection for RNA expression analyses. **b** qRT-PCR for intestinal (*cldn15la*) and Notch signaling pathway markers (*her6* and *her9*). Each dot represents a pool of 10 separated intestines and body remnants collected from 4 independent experiments. **c** Representative images of *Tg(tp1:GFP)* in WT or *il10*-Mut genetic backgrounds. Scale bar = 200 μm. **d** Quantification of the number of *tp1*:GFP^+^ cells in the intestines of WT or *il10*-Mut individuals. Each dot corresponds to 1 larva. Two independent experiments were performed. **e** Schematics for the rescue experiments in which *il10*-Mut larvae are exposed to the Notch activator Yhhu-3792. **f** Representative pictures of alcian blue stainings from 5dpf WT and *il10*-Mut larvae after treatment with Yhhu-3792. Scale bar = 100 μm. **g** Quantification of the ab-stained area after Yhhu-3792. Each dot represents individual larvae collected from two independent experiments. Independent experiments are shown with different symbol shapes in **b**, **d** and **g**. Two-way ANOVAs with Fisher’s LSD multiple comparisons tests were performed in **b** and **g**, while a two-tailed t-test was performed in **d** (*p < 0.05; ***p < 0.001).

To gain insights into the potential mechanisms by which *il10* modulates goblet cell numbers in zebrafish larvae, we analyzed the activity of signaling pathways involved in goblet cell differentiation, such as Notch,^18^ RAR,^28^ and ARP/ASCL factors.^43,44^ For this purpose, we dissected intestines from 5dpf WT and *il10*-Mut larvae (Fig. 5a) and we analyzed the expression of Goblet cell markers and key target genes for the above-mentioned signaling pathways by qRT-PCR. We did not observe differences in the expression of the RAR signaling target gene *cyp26a1*, the ARP/ASCL-associated genes *atoh1a* and *ascl1a*, nor in the expression of the goblet cell markers *agr2* and *muc2.1* (Fig. S5a). In addition, we did not detect differences in the expression of *olfm4.2*, ortholog for the mammalian stem cell marker Olfm4, nor in the expression of the teleost intestinal progenitor marker *sox9b* (Fig. S5b). However, we found a reduction in the intestinal expression levels of *her6* and *her9*, both Notch target genes described as the ortholog of human *HES1* and *HES5*, respectively (Fig. 5b). To confirm our observations, we combined the *il10*-Mut fish with the Notch activity reporter *Tg(tp1:EGFP),*^45^ which detects Notch-responding cells in the larval intestinal tract.^46^ In vivo imaging of the intestines of 5dpf *Tg(tp1:EGFP)* larvae in a WT and *il10*-Mut background proved that the number of GFP^+^ cells in the intestines of *il10*-Mut reporters was lower compared to WT (Fig. 5c, d), thus demonstrating an impaired Notch activity in *il10*-Mut larval intestines. In mammals, intracellular IL-10 downstream signaling relies predominantly on the activity of the transcription factor Stat3,^47^ and the zebrafish intestine was shown to contain Stat3-responsive cells with progenitor-like features.^48^ To determine whether Stat3 activity is impaired in the intestines of *il10*-Mut larvae, we crossed *il10*-Mut with the Stat3 activity reporter *Tg(7xStat3:EGFP).*^48^ Imaging and quantification of the number of intestinal GFP^+^ cells showed no differences between WT and *il10*-Mut larvae (Fig. S5c, d), suggesting that Stat3 signaling is not affected in *il10*-Mut larvae.

It has been previously shown that Notch signaling controls secretory cell differentiation in a time-specific manner, particularly during the phase of intestinal proliferation and polarization between 64–74 hpf.^49^ We validated these findings by using the γ-secretase/Notch inhibitor DAPT at 2 different time points: from 48 to 72 hpf (hereinafter referred to as 2–3 dpf) and from 72 to 120 hpf (referred to as 3–5 dpf, Fig. S6a). We found that Notch inhibition during 2-3dpf led to an increased ab-stained area in WT larvae, while 3–5 dpf inhibition did not modulate ab levels (Fig. S6b, c). Additionally, we tested goblet cell abundance after inhibiting Stat3 signaling with the Jak2/Stat3 inhibitor AG-490^48^ and found a reduced ab-stained area in AG-490-treated compared to control larvae, at both 2-3dpf and 3–5dpf time points (Fig. S6d–f). These results are opposite to our observations in *il10*-Mut larvae and suggest that Stat3 signaling is unlikely to play a role in the goblet cell hyperplasia observed in *il10*-Mut larvae. Therefore, we sought to determine whether boosting Notch activity between 2–3dpf restores ab-stained area to normal levels in *il10*-Mut larvae. For this purpose, we took advantage of the Notch activator Yhhu-3792^50^ and tested its activity in zebrafish larvae. Incubation of WT larvae with 1 μM of Yhhu-3792 was sufficient to upregulate the expression of *her6* transcripts (Fig. S7a, b). We then analyzed goblet cell abundance in WT and *il10*-Mut larvae exposed or not to 1 μM of Yhhu-3792 at 2-3 dpf (Fig. 5e). Yhhu-3792 treatment of *il10*-Mut larvae resulted in reduced ab-stained area compared to vehicle-treated *il10*-Mut and comparable to WT larvae (Fig. 5f, g). As expected, Yhhu-3792 treatments in *il10*-Mut larvae at 3–5dpf did not result in changes in the ab-stained area (Fig. S7c–e). These results indicate that the increased abundance of goblet cells in *il10*-Mut larvae is restored by activating Notch signaling in a specific temporal window.

### IL-10 enhances Notch signaling and restricts mucin production in mouse small intestine organoids

**Fig. 6:**
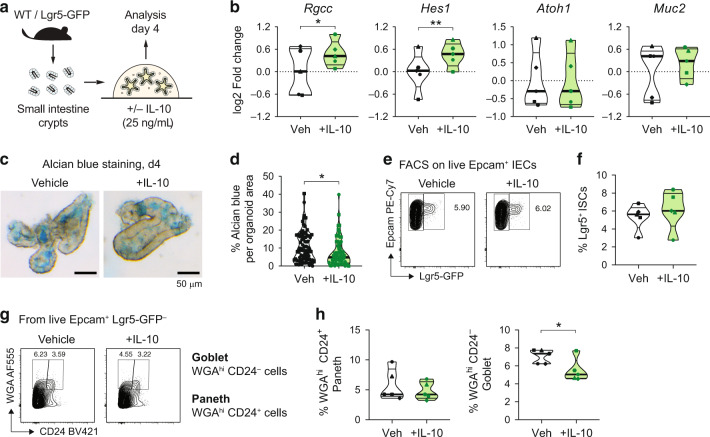
Enhanced Notch signaling and decreased goblet cell frequencies in mouse small intestine organoids treated with IL-10. **a** Schematics for mouse SI organoid cultures. Organoids were grown in ENR media (containing EGF, Noggin, and R-Spondin) supplemented with recombinant murine IL-10 or BSA (Vehicle control). **b** RNA expression analysis by qRT-PCR from organoids treated with IL-10. Each dot represents SI organoids generated from an individual mouse (*N* = 5 independent mice). **c** Alcian blue staining of SI organoids in vehicle- and IL-10-treated organoids. Scale bar = 50 µm. **d** Percentage of the ab-stained area from the total organoid area. Dots represent single organoids imaged from 2 independent experiments/mice. **e** Flow cytometry analysis of organoids generated from SI crypts of Lgr5-GFP mice. Representative contour plots showing the percentage of intestinal stem cells (Epcam^+^ Lgr5-GFP^+^ cells) are shown. **f** Frequency of Lgr5^+^ intestinal stem cells (ISCs) after IL-10 treatments. Dots represent pools of organoids collected from 5 different mice. **g** Representative contour plots of WGA/CD24 stainings on disaggregated organoids treated with IL-10. **h** Frequencies of goblet cells (Epcam^+^ Lgr5-GFP^-^ WGA^hi^ CD24^-^) and paneth cells (Epcam^+^ Lgr5^-^ WGA^hi^ CD24^+^) from vehicle and IL-10-treated SI organoids. *N* = 5 independent mice. Independent experiments are represented by different symbol shapes in **b**, **d**, **f** and **h**. Conventional two-tailed *t*-test was performed in **d**, whereas paired two-tailed *t* tests were performed in **b**, **f** and **h** (*p < 0.05, **p < 0.01).

To address whether our findings in zebrafish can be translated to mammals, we used a 3D murine small intestine (SI) organoid system which models key features of intestinal differentiation and development in vitro.^51^ We isolated crypts from wild-type C57/Bl6 mice and we cultured them in EGF-, Noggin-, and R-Spondin-containing media (ENR medium; see methods) supplemented or not with recombinant murine IL-10 for 4 days (Fig. 6a). Crypt domains per organoid and organoid areas were comparable between treatments on day 4 (d4) of treatment (Fig. S8a-c). However, transcriptional analyses of d4 organoids showed that IL-10 treatment increased the expression of the stem cell-related gene *Rgcc* and the Notch target gene *Hes1*, compared to vehicle-treated organoids (Fig. 6b). Similar to zebrafish, we did not observe differences in the expression of the secretory cell differentiation driver *Atoh1* and the goblet cell marker *Muc2* (Fig. 6b), nor in additional markers for intestinal stem cells (*Olfm4*) and other secretory cell types including enteroendocrine (*Chga*), tuft (*Dclk1*) and Paneth cells (*Lyz1*) (Fig. S8d). Further, we did not find differences in the expression levels of the intestinal differentiation marker *Krt20* and the Wnt member gene *Wnt3* (Fig. S8d). To analyze goblet cells abundance in control and IL-10-treated organoids, we modified the zebrafish ab staining protocol to perform whole-mount staining in matrigel-embedded SI organoids (see methods). IL-10-treated SI organoids exhibited a reduced percentage of ab staining per organoid area when compared to vehicle-treated organoids (Fig. 6c, d). To confirm our findings and expand our analysis to additional intestinal cell populations, we performed flow cytometry analysis of d4 SI organoids generated from Lgr5-GFP mice^52^ and stained with Epcam (Fig. S9a, b), together with WGA and CD24 to detect intestinal stem and secretory cells populations, as previously described.^53,54^ IL-10 treatments did not affect the frequency of organoid ISCs (defined as live Epcam^+^ Lg5-GFP^+^ cells, Fig. 6e, f). However, IL-10 treatments reduced specifically the frequencies of goblet cells (defined as live Epcam^+^ Lgr5-GFP^-^ WGA^hi^ CD24^-^ cells), while Paneth cells (live Epcam^+^ Lgr5-GFP^-^ WGA^hi^ CD24^+^ cells) remained unaltered (Fig. 6g, h). Further, immunofluorescence stainings of d4 SI organoids with WGA and the Paneth cell marker Lysozyme (Lyz)^55^ confirmed the presence of a group of WGA^+^ Lyz^-^ cells corresponding to goblet cells (white arrows on Fig. S9c), and its reduction after IL-10 treatments (Fig. S9d). Therefore, these results conclude that IL-10 signaling in intestinal epithelial cells modulates goblet cell frequencies across vertebrates through the activation of Notch signaling.

## Discussion

How IL-10 signaling controls intestinal immune homeostasis has been an active area of research over the past two decades. However, due to the viviparous nature of the sample sources such as humans and mice, many of these studies were performed in adult specimens, and studies understanding the role of cytokines in intestinal development remain elusive. The ex utero embryonic development and the transparency of the zebrafish larva made it an ideal model to investigate the role of cytokines in the development of intestinal mucosal immunology. Here, we combined zebrafish and mouse organoids to demonstrate that IL-10 signaling controls goblet cell homeostasis in a mechanism involving Notch signaling. Thus, we provided additional evidence showing that cytokines can act beyond leukocytes to orchestrate tissue development and that zebrafish is an excellent model organism to investigate the development and maturation of intestinal mucosal barrier functions.

The relationship between IL-10 signaling and goblet cell function has been a matter of study over the past years. An early study showed that *Muc2* was predominantly overexpressed but aberrantly sulfated in the colon of IL-10 deficient mice.^56^ This was further addressed in a later report, which showed alterations in the colonic mucus layer of IL-10 deficient mice, specifically a thicker adherent mucus layer which was however more permeable to bacteria^57^. In line with a role in orchestrating epithelial homeostasis, IL-10R signaling disruption specifically in intestinal epithelial cells (IECs) skewed IEC differentiation towards goblet cells over absorptive enterocytes.^14^ Moreover, using mouse organoids treated with recombinant IL-10 it was proposed that goblet cell modulation could be a consequence of altered intestinal stem cell (ISC) maintenance and/or differentiation.^13^ However, how IL-10 signaling might control goblet cell homeostasis, whether IL-10 signaling acts directly on ISC, and if such cytokine-IEC crosstalk occurs early in development remained elusive. Using zebrafish, our study proposes that IL-10 signaling regulates intestinal goblet cell homeostasis early in intestinal development in a mechanism involving Notch signaling and without affecting the ISC pool. The latter was investigated in murine organoids and zebrafish, using the Lgr5 reporter mice and zebrafish markers such as *olfm4.2*, the ortholog of the mammalian stem cell marker *OLFM4* and a potential marker for intestinal stem cells in adult zebrafish.^58^ In addition, we did not observe differences in the teleost intestinal progenitor marker *sox9b,*^48,59,60^ nor in the proportion of absorptive neutral red^+^ lysosome-rich enterocytes (LREs), suggesting that *il10* deficiency in zebrafish leads to a specific increase in goblet cells in the larval mid intestine.

We found a temporal correlation between intestinal *il10* expression and Notch activity in controlling secretory cell differentiation. Further, our data highlighted IL-10 signaling as a potential upstream regulator of Notch activity in the zebrafish intestine, which helped to explain the increase of goblet cells observed in *il10*-Mut larvae. Although the inhibition of Notch activity at the peak of intestinal *il10* expression(2-3dpf) recapitulated the phenotypes observed in *il10*-Mut larvae, rescue experiments are needed to directly link Notch as a downstream effector of IL-10 signaling. To rescue Notch activity, a previous study used transgenic animals that overexpressed the Notch intracellular domain (NICD), leading to a permanent activation of Notch signaling.^42^ In our approach, we used a novel synthetic Notch signaling activator Yhhu-3792^50^ which allowed us to rescue Notch signaling specifically at the 2-3dpf time window. Since intestinal ab^+^ goblet cells were restored to WT levels in *il10*-Mut after Yhhu-3792-mediated Notch activation at 2-3dpf, our data strongly suggest that early expression of *il10* is key in controlling goblet cell homeostasis in zebrafish larvae through the regulation of Notch activity. Although Stat3 could serve as a potential link between IL-10 and Notch signaling, as previously reported on monocytes,^61^ our data suggest a Stat3-independent mechanism linking both signals. An alternative mechanism could be PI3K/Akt signaling, previously described to be activated by IL-10,^62,63^ or other members of the Stat family which can be activated by IL-10, such as Stat1 and Stat5.^47^ However, further studies will be required to find the exact mechanisms which might explain the connection between *il10* and Notch signaling in the intestine of zebrafish.

Our results from mouse small intestine (SI) organoids revealed that the IL-10-Notch axis is also present in mammals and further confirms its independence from the microbiota. Although we did not find differences in the transcript levels for secretory cell markers in organoids, it is possible that these changes may be visible at a protein level, as observed in zebrafish. In addition, SI organoids do not show elevated numbers of goblet cells under normal sterile growth conditions, thus the addition of inducers of goblet cell differentiation, such as type 2 cytokines or Notch inhibitors^64^ would be needed to potentially visualize its regulation by IL-10. Nevertheless, our results from SI organoids match previous findings from single-cell transcriptomic analysis of mouse intestinal organoids, which showed an increased *Rgcc* expression after IL-10 treatments.^13^ Given that both *Rgcc* and *Hes1* are used as markers for intestinal progenitors in mice,^65^ there is the possibility that IL-10 is modulating the intestinal stem cell (ISC) pool. However, we did not observe changes in Epcam^+^ Lgr5-GFP^+^ ISCs in our organoid settings after IL-10 treatments. Therefore, the decreased average in the ab-stained area from IL-10-treated organoids, as well as the decreased frequency of Epcam^+^ Lgr5-GFP^-^ WGA^hi^ CD24^-^ cells observed by FACS, indicate that goblet cell homeostasis is specifically modulated by IL-10 in mice. Deeper analyses, focusing on specific intestinal cell populations, are required to better elucidate if IL-10 signaling impacts the specification of intestinal precursors to a secretory lineage or acts directly on goblet cells to control its function.

Our study might have implications for a better understanding of pediatric IBD, as mutations in IL-10 signaling pathway-associated genes are frequent in very-early onset (VEO) IBD cases, and dysregulation of mucus biosynthesis and goblet cell numbers are classic features of IBD. Thus, it is likely that defective IL-10 signaling may contribute to the severity of VEO-IBD by affecting early intestinal epithelial cell development, which may impact subsequent processes such as microbiota colonization. The fact that IL-10 can act beyond immune responses and directly control the differentiation of IECs raises several points to be considered in clinical studies. First, it suggests that aberrant immune responses are not the only driving cause for the development of VEO-IBD in patients carrying mutations in IL-10 signaling-related genes, which explains their resistance to immunosuppressive therapies^66^ and improving defective intestinal development and differentiation might also be considered for therapeutics.^67^ Second, although alterations in mucus and goblet cells are observed in active VEO-IBD cases,^7,68^ there is no clear information about the intestinal cell composition before the onset of the disease and with no active inflammation, which could change the view on the causal factors and the physiological consequences of IBD pathogenesis. Third, despite the lack of clinical evidence directly linking IL-10 signaling, goblet cells, and VEO-IBD, data from experimental models support this idea, and further investigation needs to be conducted to better understand how these factors play a role in the initiation of the disease.

Thus, we propose the existence of an IL-10-Notch axis in the early development of intestinal epithelial cells which regulates mucin production, goblet cell differentiation, and is conserved across vertebrates.

## Material and methods

### Animals

Zebrafish (*Danio rerio*, AB strain) were reared and kept in the Karolinska Institutet (KI) Zebrafish Core Facility and the Institut Curie Animal Facility, according to standard protocols. Ethical permits used for zebrafish husbandry were Nr 5756/17 (to Eduardo Villablanca), 14049/19 (to KI Zebrafish Core Facility), and APAFiS #21197-2019062521156746-v2 (To Pedro Hernandez). Zebrafish embryos were collected by natural spawning of adults and were kept at 28 °C in E3 water. The zebrafish line *TgBAC(cldn15la-GFP)*^pd1034,^ ^36^ referred to in the text as *Tg(cldn15la:GFP)*, was provided by Prof. Michael Bagnat (Duke University Medical Center, USA); the line *Tg(Tp1bglob:eGFP)*^um14,^ ^45^ referred in the manuscript as *Tg(tp1:EGFP)* was kindly provided by Prof. Olov Andersson (Department of Cell and Molecular Biology CMB, Karolinska Institutet, Sweden); and the line *Tg(7xStat3-Hsv.Ul23:EGFP),*^48^ referred in the manuscript as *Tg(7xStat3:EGFP)*, was kindly provided by Prof. Francesco Argenton (Università degli Studi di Padova, Italy).

Wild-type mice (*Mus musculus*, C57/Bl6 strain) were purchased from Charles River Laboratories or Taconic and were kept in the Comparative Medicine facility from Karolinska University Hospital (AKM). The transgenic strain *Lgr5-EGFP-IRES-creERT2,*^52^ referred to in the text as Lgr5-GFP, was maintained in a C57/Bl6 background at the Comparative Medicine facility from Karolinska Institutet (KM-B). All mice used for experiments were 6–10 weeks old. Animals were handled according to protocols approved by the Stockholm Regional Ethics Committee (Nr 3227-2017 and 6778-2020). All experiments were performed following the national and institutional guidelines and regulations.

### Isolation of fluorescent intestinal epithelial cells from zebrafish larvae

To acquire the intestinal epithelial population, approximately 100 of 6dpf *TgBAC(cldn15la:GFP)* zebrafish larvae were collected for the FACS experiment, then intestines were dissected and placed into PBS on ice with a maximum dissection time of 2 h. Intestine dissociation was performed using TrypLE Express (Gibco, Cat. No. 12605028) for 1 h at 37 °C, pipetting up and down every 10 min to support digestion. Digested samples were spun at 1500 g for 5 min at 4 °C and washed twice with PBS 1× before being resuspended together with PBS 1× and 10% FBS (fetal bovine serum). Filtered cells were immediately subjected to FACS at Institut Curie Flow Cytometry Platform using the Sony SH800 Cell Sorter. Dead cells were excluded from analysis using the combination of Calcein Blue (Invitrogen, Cat. No. 65-0855-39) and Propidium Iodide viability stains (Sigma-Aldrich, Cat. No. P4864). Non-transgenic and single transgenic controls (pools of 10 dissected guts) were prepared as above and used for gating and compensation. RNA isolation was done using on average 30,000 GFP^+^ or GFP^−^ sorted cells with the Single-Cell RNA Purification Kit from Norgen Biotek Corp and reverse transcribed using Superscript IV Reverse Transcriptase (Life Technologies, Cat. No. 18090050) following the manufacturer’s instructions.

### Whole-mount in situ hybridization in zebrafish larvae

Portions of the coding sequences for the ZFIN-annotated orthologs of the human *IL10RA* (Gene name: *il10ra*, Gene ID: 777651, ENSEMBL ID: ENSDARG00000100383) and *IL10RB* genes (Gene name: *il10rb*, Gene ID: 619391, ENSDARG00000078042) were amplified by PCR using the primers from Table S1, and subsequently cloned into a pCRII-TOPO vector (Invitrogen, Cat. No. 450640). Probe and control RNA sequences were synthesized in vitro by using T7 and SP6 RNA polymerases and subsequently purified by Lithium chloride (LiCl) precipitation. Whole-mount in situ hybridizations were performed in 3 dpf and 5 dpf zebrafish larvae as previously described^30^ with the following modifications. Permeabilization was done by treating fixed larvae with 10 μg/mL of Proteinase K (Qiagen, Cat. No. 19131) prepared in PBS supplemented with Tween-20 0.1% and DMSO 1% for 30 minutes at room temperature. Pre-hybridization was done at 60 °C for at least 3 h in Hybridization media (HM, Formamide 50%, Sodium citrate solution 5×, citric acid 9.2 mM, Heparin 50 μg/mL, RNAse-free yeast tRNA 500 μg/mL). Probe hybridization was done using 500 μL of HM containing 4 ng/mL of either probe or control RNA, and samples were incubated overnight at 60 °C. Anti-dig-AP antibody (Roche, Cat. No. 1109327493) was diluted 1:3000 in PBS supplemented with Tween-20 0.1%, sheep serum 2%, and bovine serum albumin (BSA) 2 mg/mL. Development of probe hybridizations was done by diluting NBT-BCIP stock solution (Roche, Cat. No. 11681451001) 1:50 in alkaline phosphatase buffer (Tris-HCl 100 mM pH 9.5, MgCl_2_, NaCl, Tween-20 0.1%). After 3 h of developing, samples were washed extensively with PBS plus Tween-20 0.1%, fixed in PFA 4%, washed again in PBS Tween-20 0.1%, and subsequently dehydrated in Methanol and stored at −80 °C for at least one night, to allow clearing of background signal. Larvae were rehydrated, transferred to Glycerol 85%, and imaged using a Nikon SMZ25 stereoscope equipped with a DS-Fi3 camera.

### Generation of *il10*-Mut zebrafish lines

The zebrafish coding sequence for the ortholog of the human *IL10* gene (Gene name: *il10*, Gene ID: 553957, ENSEMBL ID: ENSDARG00000078147)^31^ was targeted by CRISPR/Cas9^37^ with specific sgRNAs (Table S1). For the maintenance of wild-type and *il10* mutant stocks, zebrafish embryos coming from the incross of heterozygous *il10* individuals were raised, and genotyping was performed once they reached adulthood by fin-clipping a portion of the tail fin. The *il10*^uu1751^ mutation created a restriction enzyme site for EcoNI, which is absent in wild-type fish. Carriers of the *il10*^uu1751^ mutation were screened by PCR followed by enzymatic digestion with EcoNI. Zebrafish carrying the *il10*^*uu1762*^ mutation were screened by PCR using combinations of primers in which the forward primer was specific for either the WT or mutant sequence. Primers used for genotyping are shown in Table S1.

### Cloning of the zebrafish *il10* and *il17a/f3* genes and rescue experiments

The coding sequences for the zebrafish *il10* and *il17a/f3* genes were amplified by PCR using specific primers for both genes (Table S1) and then cloned in a pCR2.1-TOPO vector (Invitrogen, Cat. No. 450641). In vitro- transcribed mRNA was synthesized using the T7 mMessage mMachine kit (Invitrogen, Cat. No. AM1344), following the manufacturer’s guidelines. For injections, 1nL of 200 ng/μL of in vitro-transcribed mRNA was co-injected with 0.5% of Rhodamine-Dextran MW10000 (Invitrogen, Cat. No. D1824) in 1-cell stage wild-type or *il10*-Mut embryos. Embryos injected with 0.5% Rhodamine-dextran were used as control. At 2dpf, Rhodamine-positive embryos were selected and used for subsequent analysis.

### Body-intestine isolation in zebrafish larva

Larvae from 3 dpf to 5 dpf were euthanized by overdosing them with MS-222 and intestines were isolated mechanically with the use of needles to pierce the tissue and extract the intestines. Around 10 intestines and their respective bodies were collected for analysis.

### RNA extraction and qRT-PCR from zebrafish samples

For zebrafish samples, up to 10 tissue samples (i.e. whole larvae, intestines, or body carcasses) were pooled and collected in Trizol reagent (Invitrogen, Cat. no. 10296010), and tissues were homogenized by pipetting the samples repeatedly through 23G and 27G needles. Total RNA was extracted following Trizol manufacturer’s instructions. Synthesis of cDNA was performed using the iScript cDNA synthesis kit (BioRad, Cat. No. 1708841). Quantitative PCR was performed using iTaq Universal SYBR Green supermix (BioRad, Cat. No. 1725124), as previously reported.^69^ Primers used for zebrafish qRT-PCR analysis are found in Table S2.

### Bleach and antibiotic treatments in zebrafish larvae

Zebrafish embryos between 24–28 hpf were treated with chlorine hypochlorite (0.04%) for 5 minutes and then washed twice with sterile E3 medium for 5 min. After washing, embryos were left in sterile E3 medium containing Ampicillin 100 μg/mL and Kanamycin 5 μg/mL and placed at 28 °C in isolated containers. Media was renewed every day in sterile conditions until the day of sample collection. For the quantification of bacterial loads, genomic DNA from 2 larvae per condition was extracted by (heat-basic), and PCR against 16S All Bacteria^30^ and zebrafish gDNA (for *il23r* gene) was performed. PCR products were run in a 2% agarose gel, and band intensities were measured in Fiji/ImageJ (NIH).

### Alcian blue, neutral red stainings and quantifications in zebrafish larvae

Stainings for Neutral red (Sigma, Cat. No. N4638) and Alcian blue (Sigma, Cat. No. B8438) in 5dpf zebrafish larvae were performed as previously described.^70^ Alcian blue-stained larvae were additionally treated with H_2_O_2_ 1.5%/KOH 0.5% to remove pigments before imaging. Larvae were mounted in a lateral position using 1% low-gelling point agarose (Sigma, Cat. No. A9414), and RGB images were acquired using a Nikon SMZ25 stereoscope equipped with a DS-Fi3 camera. Images were cropped to keep the mid-intestine section. Automatic unbiased analyses of the Alcian blue-stained area in the intestine of individual larvae were performed in Fiji/ImageJ software (NIH) using “Colour Deconvolution 1.7” and selecting the “Alcian blue & H” vector to identify the Alcian blue-stained area. For the automatic detection of the Neutral red-stained area, the function “Color Threshold” was used. The length of the alcian blue- and neutral red-stained regions were measured manually in randomized images.

### Immunofluorescence staining in zebrafish larvae

Immunostaining was performed on whole larvae at 5 days post fertilization. Paraformaldehyde at 4% was used to fix zebrafish larvae overnight at 4 °C. The samples were then washed with distilled water. For whole-mount 2F11 immunostaining, fixed larvae were permeabilized with cold 100% acetone for 20 min at 4 °C before being washed three times with PBST (PBS supplemented with 0,5% Triton-X-100). Samples were further permeabilized with 1 mg/mL Collagenase from *Clostridium histolyticum* (Cat. No. C2139) for 2 h at room temperature. Samples were then washed with PBST and blocked with 10% of FBS/PBST at room temperature for more than 2 h. The primary mouse monoclonal 2F11 antibody (1:200; Abcam, Cat. No. ab71286) was diluted in blocking solution and incubated at 4 °C for more than 24 h. Following primary antibody incubation, the samples were washed with PBST solution and incubated for at least 2 h with secondary anti-mouse Alexa Fluor 488 antibody (1:500; Invitrogen, Cat. No. A11001) at room temperature in the dark. For whole-mount WGA staining, larvae were washed in distilled water for 1 h at room temperature, permeabilized with cold 100% acetone form 20 minutes at −20 °C, and washed with PBST. Larvae were incubated in Alexa Fluor 555-conjugated Wheat Germ Agglutinin (1:5000 from 5 mg/mL stock; Invitrogen, Cat. No. W32464) overnight at 4 °C and washed extensively with PBST. Imaging was performed using the Zeiss Axio Zoom.V16 stereoscope or the Zeiss LSM800 confocal microscope and analyzed with ImageJ software.

### Chemical treatments in larval zebrafish

At 2 days post fertilization, pools of 10 larvae per milliliter were exposed to the Notch/γ-secretase inhibitor DAPT (50 μM; Tocris, Cat. No. 2634), the Jak2/Stat3 inhibitor Tyrphostin AG-490 (50 µM; Sigma-Aldrich, Cat. No. T3434), or the Notch activator Yhhu-3792 (Tocris, Cat. No. 6599), or. As a control for Notch activity induced by Yhhu-3792, RNA was extracted from treated larvae and the expression levels of the Notch downstream gene *her6* were measured by qRT-PCR.

### Fluorescent reporter imaging and analysis

The fluorescent reporters for Notch activity *Tg(tp1:GFP)* and Stat3 activity *Tg(7xStat3:EGFP)*, in either wild-type or *il10*^uu1751^ genetic background, were imaged at 5dpf in an SMZ25 stereoscope equipped with a CoolLed laser set and a DS-Fi3 camera (Nikon), focusing on the intestinal region. Z-stacks were merged in Fiji/ImageJ software, and the number of GFP^+^ cells in the intestinal region was counted manually in Fiji/ImageJ.

### Dissection, sectioning, and staining of adult zebrafish intestines

Zebrafish older than 1 year of age were euthanized with an overdose of MS-222. Intestines were collected and fixed in neutral buffered formalin (Sigma, Cat. No. HT-501128) overnight at room temperature, and subsequently transferred to ethanol 70% for at least 24 h. Tissues were dehydrated and embedded in paraffin, and 10 µm sections were collected and stained with alcian blue. Briefly, tissue sections were deparaffinized and rehydrated to PBS, and incubated in 3% alcian blue solution (Sigma) for 5 min. After staining, tissues were counterstained with nuclear fast red (Sigma, Cat. No. N3020). Images were acquired in a Nikon SMZ25 stereoscope and quantifications of goblet cells per villus area were performed using QuPath 0.2.3 (University of Edinburgh, UK).

### Mouse intestinal organoid cultures

Mouse organoids were generated from crypts derived from the entire small intestine (SI). Briefly, SIs from WT mice were collected and flushed with PBS, cut opened longitudinally, and subsequently cut into five pieces of similar size. Tissue pieces were placed in PBS and were vigorously shaken to remove mucus. Tissue pieces were then transferred to cold PBS-EDTA 10 mM and incubated for 1 h in ice. After incubation, SI villi were removed by gentle scraping of the luminal side using two glass slides. Then SI crypts were scraped from the tissue pieces by applying stronger pressure with the glass slides and collected in recipient tubes filled with cold PBS. Collected crypts were centrifuged for 5 min at 300 × *g* and 4 °C, and then quantified using an upright microscope by placing 10 µL of crypt solution on a glass slide. The basic culture medium (ENR) contained advanced DMEM/F12, 1× penicillin/streptomycin, 1x Glutamax (Thermo Fisher Scientific), 10 mM HEPES (Thermo Fisher Scientific), 1x B27 supplement (Life Technologies, Cat. No.17504044), 1× N2 supplement (Life Technologies, Cat. No. 17504048), 1mM N-acetylcysteine (NAC, from Sigma, Cat. No. A9165) and was supplemented with 50 ng/mL of recombinant murine epidermal growth factor (EGF, from R&D, Cat. No. 2028-EG), 250 ng/mL recombinant murine R-Spondin (R&D, Cat. No. 3474-RS) and 100 ng/mL recombinant murine Noggin (Peprotech, Cat. No. 250-38). SI crypts were resuspended in 30–40% basic culture medium with 60–70% Matrigel (Corning, Cat. No. 356231) and 20 µl containing approximately 500 crypts were plated in a pre-warmed flat-bottom 48-well plate (Sarstedt, Cat. No. 83.3923). The plate was placed at 37 °C and allowed to solidify for 15 min before 200 µl of ENR medium (containing the different stimuli) was overlaid. The medium was replaced every 2 days with fresh medium and cultures were maintained at 37 °C in fully humidified chambers containing 5% CO_2_. During the first 2 days of culture, the ENR medium was supplemented with 10 µM of the ROCK inhibitor Y-27632 (Sigma, Cat. No. Y0503). For organoid in vitro stimulation, 25 ng/mL of recombinant murine IL-10 (Peprotech, Cat. No. 210-10) diluted in 0.1% bovine serum albumin (BSA, Sigma) was added in the ENR medium for the entire duration of the organoid cultures. Control organoids were supplemented with a similar volume of 0.1% BSA vehicle. Each condition was plated in technical triplicates. On day 4 of culture, crypt domains per IL-10-treated or vehicle-treated organoids were quantified in 2–3 wells/condition Each dot in the quantification plot represents one mouse and an average of 2–3 technical replicates.

### Alcian blue stainings on mouse SI organoids

The Alcian blue protocol used for the staining of mouse SI organoids was adapted from the protocol used for whole-mount Alcian blue stainings in zebrafish. Organoids were collected after 4 days of culture, washed 5 times with cold PBS-BSA 0.1%, and replated in a new Matrigel stock in 24-well plates, to remove debris from the initial culture. After Matrigel gelification, organoids were fixed in PFA 4% for 45 minutes, washed twice with PBS-BSA 0.1%, and washed twice with acidic ethanol (70% Ethanol supplemented with 1% HCl). Organoids were stained with Alcian blue solution (Sigma, Cat. No. B8438) for 1 h at room temperature. After the incubation, stained organoids were washed extensively with acidic ethanol and were left overnight in acidic ethanol at 4 °C, to remove excessive staining from the Matrigel. Once the Matrigel became clear, organoids were washed with PBS-BSA 0.1% and imaged using the Nikon SMZ25 system.

### RNA extraction from mouse organoids and qRT-PCR

After 4 days of culture, treated organoids were harvested and Matrigel was removed by consecutive washes with cold PBS-BSA 0.1%. Cleaned organoids were resuspended in RLT-plus buffer supplemented with 1% β-Mercaptoethanol, and RNA extraction was performed using the RNeasy extraction kit (Qiagen, Cat. No. 74136), following the manufacturer’s instructions. Similar to zebrafish samples, synthesis of cDNA was performed using the iScript cDNA synthesis kit (BioRad), and quantitative PCR was performed using iQ SYBR Green mix (BioRad). Primers used for mouse qRT-PCR analysis are found in Table S2.

### Flow cytometry from organoid cell suspensions

For flow cytometry experiments, SI organoids were generated from crypts extracted from Lgr5-GFP mice. After 4 days of treatment, organoids were harvested and washed 5 times with cold PBS-BSA 0.1%. Organoids were disaggregated to single cells by incubating with TrypLE supplemented with DNase I for 5 min at 37 °C. Cells were incubated in Fc block (1:1000; Invitrogen, Cat. No. 14-0161-85) and Fixable Viability Dye eFluor 780 (1:1000; eBioscience, Cat. No. 65-0865-14) for 15 min at 4 °C, and subsequently stained with anti-Epcam PE-Cy7 (1:200; BioLegend, Cat. No. 118215), anti-CD24 BV421 (1:200; BioLegend, Cat. No. 101825) and WGA Alexa Fluor 555 (1:5000; Invitrogen) for 1 h at 4 °C. Flow cytometry was performed using an LSRFortessa flow cytometer (BD Biosciences, USA), and analysis was perfumed using FlowJo v10 (Treestar, USA).

### Immunostaining of small intestine organoids

For immunofluorescence experiments, Matrigel-embedded isolated crypts extracted from small intestines of WT mice were plated in pre-warmed 8-well 15 µm glass-bottom chambers (Ibidi, Cat. No. 80826) and cultured as described above. On day 4 of culture, organoids were fixed in PBS-buffered Paraformaldehyde 4% for 45 minutes, permeabilized with Triton X-100 0.5% for 30 minutes and treated with Glycine 0.1 M for 1 h to block free aldehyde groups. Organoids were incubated in blocking solution (Normal goat serum 5%, Triton X-100 0.25%, BSA 0.1% in PBS) for 30 min before incubating with WGA Alexa Fluor 555 (1:5000, Invitrogen) and rabbit polyclonal anti-Lysozyme Ab-1 (1:200; Thermo Scientific, Cat. No. RB-372-A) overnight at 4 °C. After 3 washes with PBS-BSA 0.1%, organoids were incubated with goat anti-rabbit Alexa Fluor 647 (1:1000; Invitrogen, Cat. No. A21244) overnight at 4 °C. On the final day of staining, organoid nuclei were stained with DAPI (1 µg/mL, Molecular Probes, Cat. No. D1306) for 30 min at room temperature, washed, and kept in PBS until imaging within 0–3 days. Images from stained organoids were acquired in a Zeiss LSM800 confocal microscope (Zeiss, Germany) and analyzed using ImageJ.

### Statistical analysis

Quantitative data were analyzed using GraphPad Prism v8.0 software (GraphPad, San Diego, CA, USA). Group comparisons were considered statistically significant when they reached a *p*-value below 0.05.

## Supplementary information

Supplementary Information
