# Supplementary material for: Interleukin-10 regulates goblet cell numbers through Notch signaling in the developing zebrafish intestine
Source: Mucosal Immunol. 2022 Jul 15;15(5):940–51. doi: 10.1038/s41385-022-00546-3 (PMC9385495; doi:10.1038/s41385-022-00546-3)
Supplement: Supplementary file 1 — Supplementary Information [file 41385_2022_546_MOESM1_ESM.pdf]

Figure S1

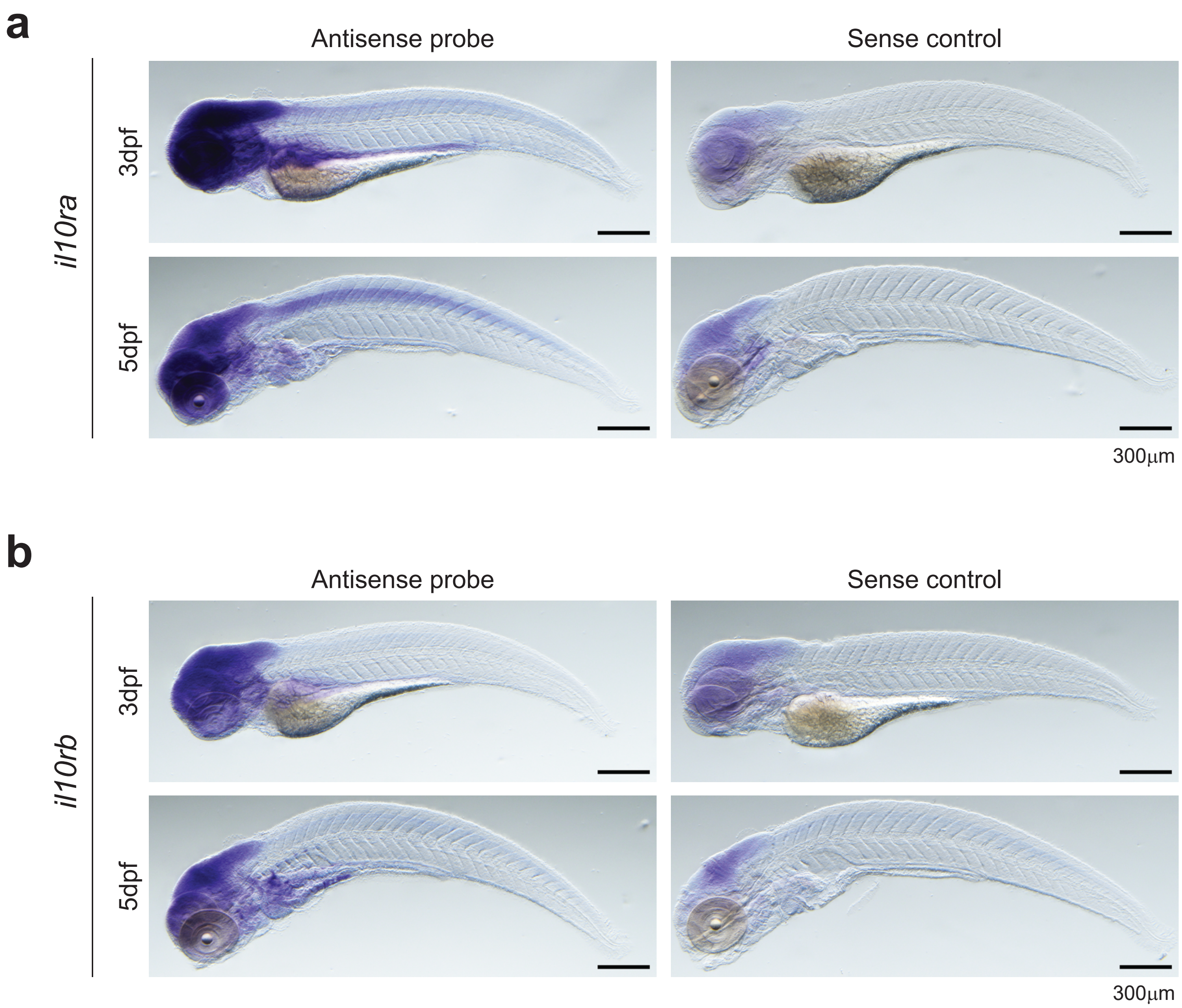

a
Antisense probe
Sense control
il10ra
3dpf
5dpf
300μm
b
Antisense probe
Sense control
il10rb
3dpf
5dpf
300μm

Figure S1. Specificity of the whole-mount *in situ* hybridizations against *il10ra* and *il10rb* in zebrafish larvae. Representative pictures of complete 3dpf and 5dpf larvae labeled with antisense probe and sense control oligos against *il10ra* (**a**) and *il10rb* (**b**). Scale bar = 300µm.

Figure S2

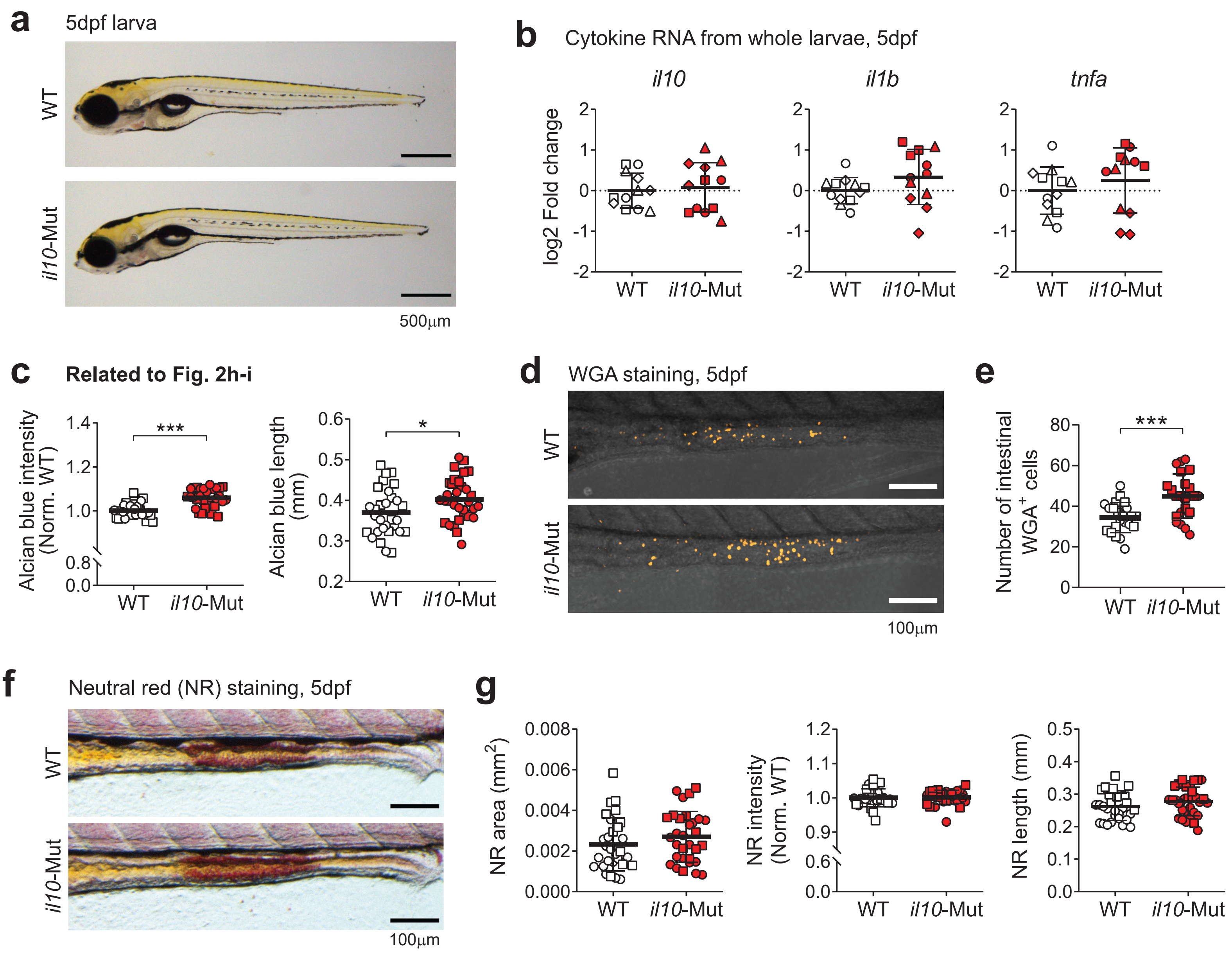

a
5dpf larva
WT
il10-Mut
500µm
b
Cytokine RNA from whole larvae, 5dpf
il10
il1b
tnfa
log2 Fold change
2
1
0
-1
-2
WT
il10-Mut
c
Related to Fig. 2h-i
Alcian blue intensity (Norm. WT)
1.4
1.2
1.0
0.8
0.0
***
Alcian blue length (mm)
0.6
0.5
0.4
0.3
0.2
*
WT
il10-Mut
d
WGA staining, 5dpf
WT
il10-Mut
100µm
e
Number of intestinal WGA+ cells
80
60
40
20
0
***
WT
il10-Mut
f
Neutral red (NR) staining, 5dpf
WT
il10-Mut
100µm
g
NR area (mm2)
0.008
0.006
0.004
0.002
0.000
NR intensity (Norm. WT)
1.2
1.1
1.0
0.9
0.6
0.0
NR length (mm)
0.5
0.4
0.3
0.2
0.1
0.0
WT
il10-Mut

Figure S2. Characterization of *il10*-Mut zebrafish larvae. (**a**) Representative images of 5dpf WT and *il10*-Mut zebrafish larvae. Scale bar = 500µm. (**b**) Whole-body expression analysis of cytokines by qRT-PCR. Each dot represents a pool of 5-10 larvae collected from 3-4 independent experiments. (**c**) Alcian blue signal intensity and length from analysis performed in Figure 2h-i. N = 2 independent experiments. (**d**) Confocal images from whole-mount WGA stainings on 5dpf WT and *il10*-Mut larvae. Scale bar = 100µm. (**e**) Quantification of the number of WGA^+ goblet cells in the mid intestines of 5dpf WT and *il10*-Mut larvae (N = 2 independent experiments). (**f**) Neutral red stainings on 5dpf WT and *il10*-Mut larvae. Scale bar = 100µm. (**g**) Area, intensity, and length measurements for the neutral red-stained regions. Each dot represents an individual larva collected from 2 independent experiments. Two-tailed t-tests were performed in **b**, **c**, **e** and **g** (*p< 0.05; ***p<0.001).

Figure S3

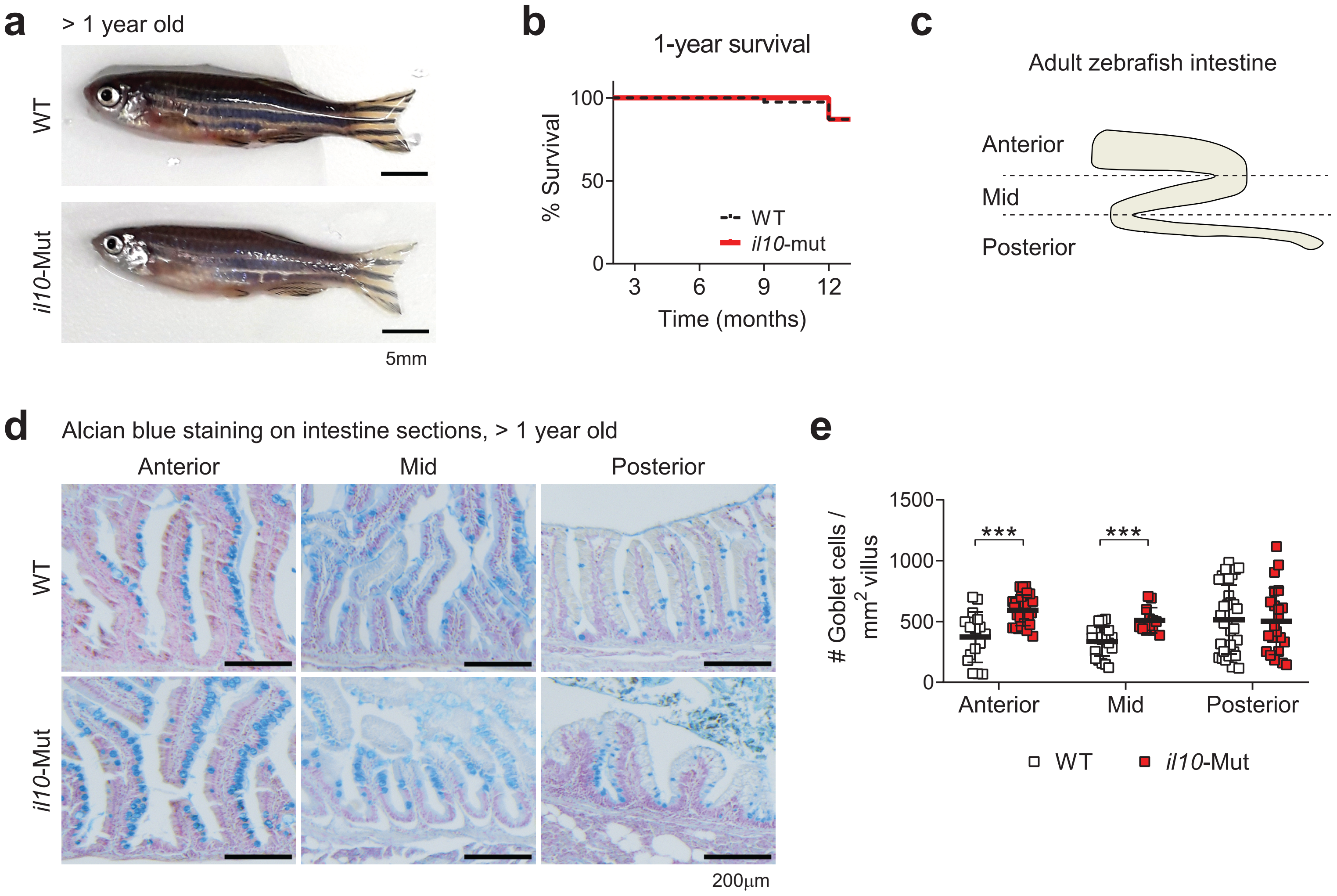
a
> 1 year old
WT
il10-Mut
5mm
b
1-year survival
% Survival
100
50
0
3
6
9
12
Time (months)
WT
il10-mut
c
Adult zebrafish intestine
Anterior
Mid
Posterior
d
Alcian blue staining on intestine sections, > 1 year old
Anterior
Mid
Posterior
WT
il10-Mut
200µm
e
Goblet cells / mm2 villus
1500
1000
500
0
***
***
Anterior
Mid
Posterior
WT
il10-Mut

Figure S3. Characterization of *il10*-Mut adults. (**a**) Images of WT and *il10*-Mut zebrafish adults. Scale bar = 5mm. (**b**) Survival of WT and *il10*-Mut zebrafish over a period of 1 year (N= 38 for WT and 39 for *il10*-Mutant, out of 2 independent breedings). (c) Representation of an adult zebrafish intestine and the sections used for analysis. (d) Representative alcian blue staining images from intestinal sections of WT and *il10*-Mut zebrafish. Scale bar = 200µm. (e) Quantification of goblet cells (ab^+ cells) from WT and *il10*-Mut intestine sections. The number of goblet cells was normalized by the area of the villi analyzed. Unpaired two-tailed t-tests were used in **e** (***$p < 0.001$).

Figure S4

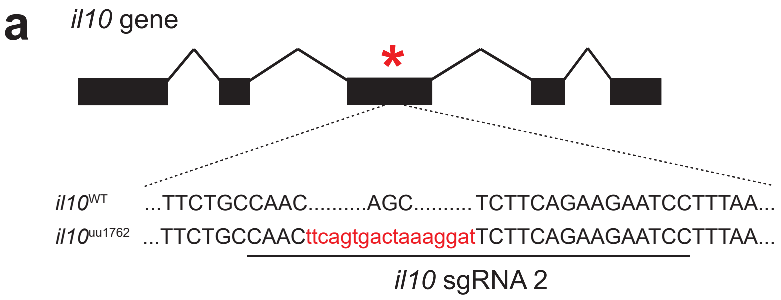

b

Il10WT

```
MIFSGVILSALLTLLLCDCAQSRRVECKTDCCSFVEGFPLRLRELRSAYK
EIQKFYESNDDLEPLLNEDIKHNINSPYGCHVMNEILHFYLETILPTALQ
KNPLKHSTTPIDSIGNIFQELKRDMVKCKRYFSCQNPFEVNSLKNSYEKM
KEKGVYKAMGELDLLFRYIEQYLASKRVKH*
```

Il10uu1762 (Il10-Mut2)

```
MIFSGVILSALLTLLLCDCAQSRRVECKTDCCSFVEGFPLRLRELRSAYK
EIQKFYESNDDLEPLLNEDIKHNINSPYGCHVMNEILHFYLETILPTSVT
KGFFRRIL*
```

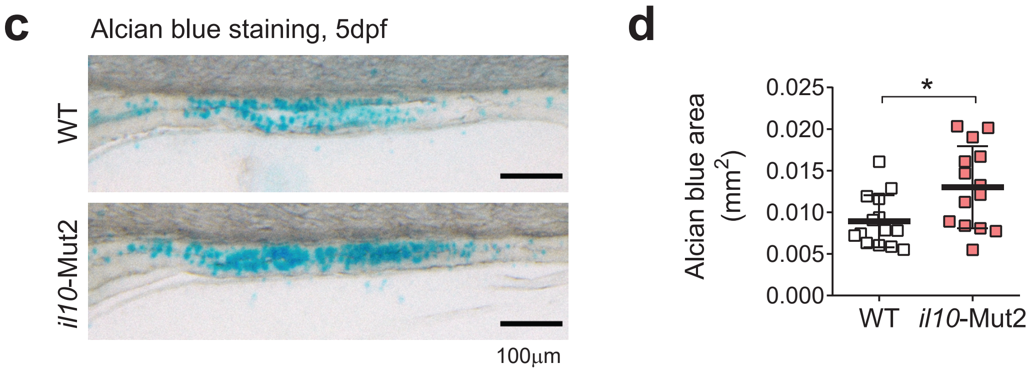

Figure S4. Increased intestinal alcian blue^{+} goblet cells in a second mutant line for *il10*. (**a**) Schematics for the second mutation generated in the zebrafish *il10* gene (*il10*^{uu1762}, +14bp) by CRISPR/Cas9. (**b**) Predicted sequences for Il10 protein in *il10*-Mut2 individuals, compared to WT. (**c**) Alcian blue stainings in the mid intestines of 5dpf WT and *il10*-Mut2 larvae. (**d**) Automatic quantification of the ab-stained area of WT and *il10*-Mut larvae. A two-tailed t-test was performed in **b** (*$p<0.05$).

Figure S5

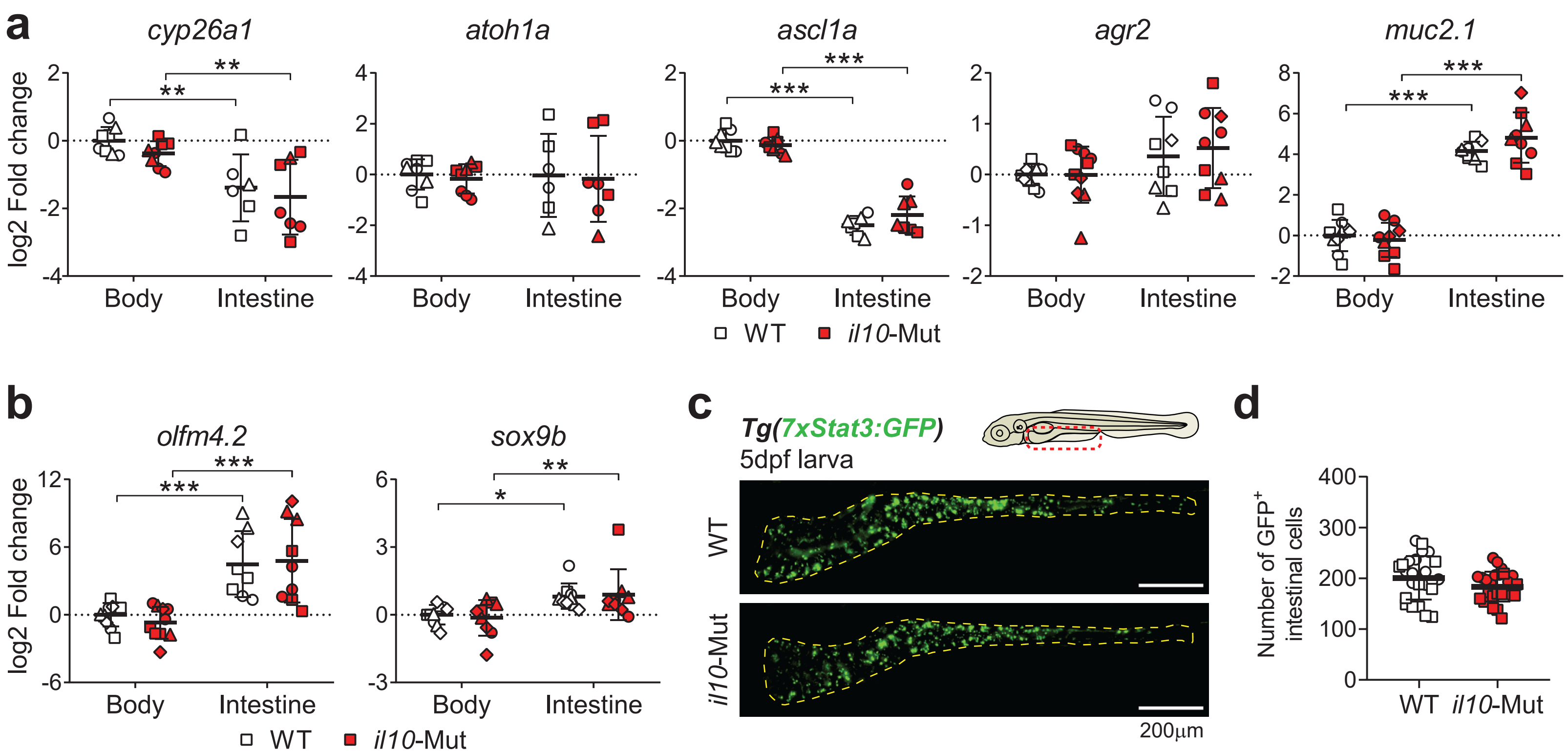
a
cyp26a1
atoh1a
ascl1a
agr2
muc2.1
log2 Fold change
Body
Intestine
**
***
WT
il10-Mut
b
olfm4.2
sox9b
*
c
Tg(7xStat3:GFP)
5dpf larva
WT
il10-Mut
200μm
d
Number of GFP+ intestinal cells

Figure S5. Body-intestine transcriptomic profile of WT and *il10*-Mut zebrafish larvae. RNA from dissected intestines and body remnants from 5dpf WT and *il10*-Mut larvae was used to perform qRT-PCR analysis against RAR, ARP/ASCL signaling pathways, and markers of goblet cells (**a**) and for the candidate markers of intestinal progenitors *olfm4.2* and *sox9b* (**b**). Each dot corresponds to RNA from a pool of 10 intestines or body remnants, collected in 3-4 independent experiments. (**c**) Representative pictures of the intestines of *Tg(7xStat3:EGFP)* in WT and *il10*-Mut genetic backgrounds. Scale bar = 200µm. (**d**) Number of GFP^+ cells in the intestines of 5dpf *Tg(7xStat3:GFP)* WT and *il10*-Mut larvae. Dots represent individual larvae collected from 2 independent experiments. Two-way ANOVAs with Fisher LSD multiple comparison tests were used in **a** and **b**, whereas a two-tailed t-test was performed in **d** (**p<0.01; ***p<0.001).

Figure S6

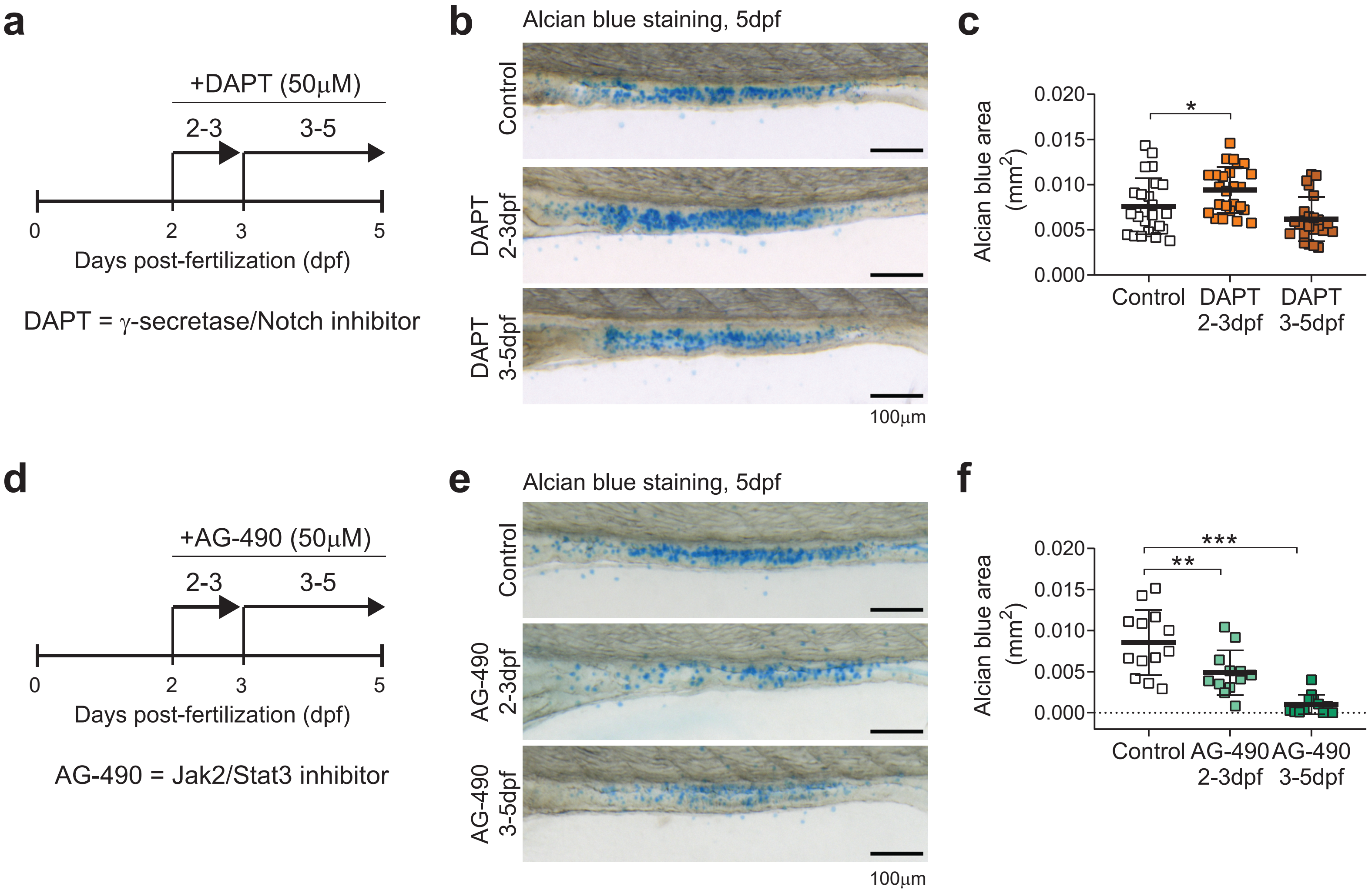
a
+DAPT (50μM)
2-3
3-5
0
2
3
5
Days post-fertilization (dpf)
DAPT = γ-secretase/Notch inhibitor
b
Alcian blue staining, 5dpf
Control
DAPT 2-3dpf
DAPT 3-5dpf
100μm
c
Alcian blue area (mm²)
0.020
0.015
0.010
0.005
0.000
*
Control
DAPT 2-3dpf
DAPT 3-5dpf
d
+AG-490 (50μM)
2-3
3-5
0
2
3
5
Days post-fertilization (dpf)
AG-490 = Jak2/Stat3 inhibitor
e
Alcian blue staining, 5dpf
Control
AG-490 2-3dpf
AG-490 3-5dpf
100μm
f
Alcian blue area (mm²)
0.020
0.015
0.010
0.005
0.000
***
**
Control
AG-490 2-3dpf
AG-490 3-5dpf

Figure S6. Early inhibition of Notch promotes alcian blue^{+} goblet cell expansion in WT larvae. (**a**) Diagram showing the times of treatments with the γ-secretase/Notch inhibitor DAPT in WT zebrafish larvae. (**b**) Alcian blue staining of DAPT-treated zebrafish larvae at 5dpf. Scale bar = 100µm. (**c**). Automatic quantifications of the ab-stained area of DAPT-treated larvae at 5dpf (1 dot = 1 larva). (**d**) Schematics for the treatment of WT larvae with the Jak2/Stat3 inhibitor AG-490. (**e**) Alcian blue staining of AG-490-treated zebrafish larvae at 5dpf. Scale bar = 100µm. (**f**). Automatic quantifications of the ab-stained area of DAPT-treated larvae at 5dpf (1 dot = 1 larva). One-way ANOVAs were performed in **c** and **f**.

Figure S7

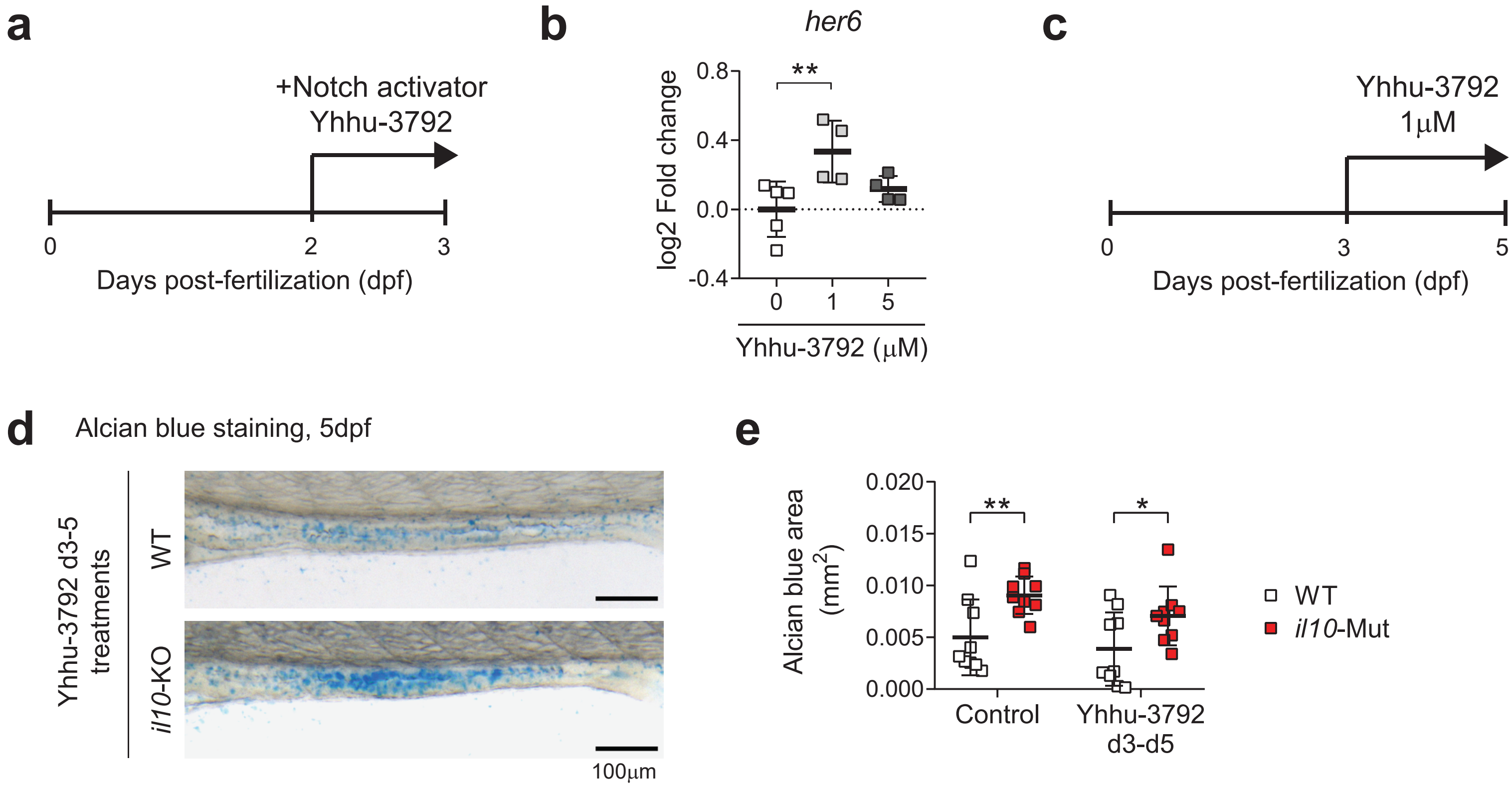

a
+Notch activator
Yhhu-3792
0
2
3
Days post-fertilization (dpf)
b
her6
log2 Fold change
0.8
0.4
0.0
-0.4
**
0
1
5
Yhhu-3792 (μM)
c
Yhhu-3792
1μM
0
3
5
Days post-fertilization (dpf)
d
Alcian blue staining, 5dpf
Yhhu-3792 d3-5 treatments
WT
il10-KO
100μm
e
Alcian blue area (mm2)
0.020
0.015
0.010
0.005
0.000
**
*
Control
Yhhu-3792 d3-d5
WT
il10-Mut

Figure S7. Validation of the Notch activator Yhhu-3792 in zebrafish larvae. (**a**) Strategy to test the activity of Yhhu-3792 in zebrafish larvae. Different doses of Yhhu-3792 were used to treat larvae between 2-3dpf. (**b**) Expression of the Notch target gene *her6* in 3dpf zebrafish larvae after treatments with different concentrations of Yhhu-3792. Each dot represents a pool of 10 larvae collected from 2 independent experiments. (**c**) Strategy to test for Notch rescue experiments in *il10*-Mut larvae between 3-5dpf. (**d**) Representative alcian blue stainings of 5dpf WT and *il10*-Mut zebrafish after treatments with Yhhu-3792 between 3-5dpf. Scale bar = 100μm. (**e**) Quantifications of the ab-stained area in the intestines of 5dpf WT and *il10*-Mut larvae after treatments. One-way ANOVA was performed in **b**, while Two-way ANOVA was performed in **e** (*p<0.05; **p<0.01).

Figure S8

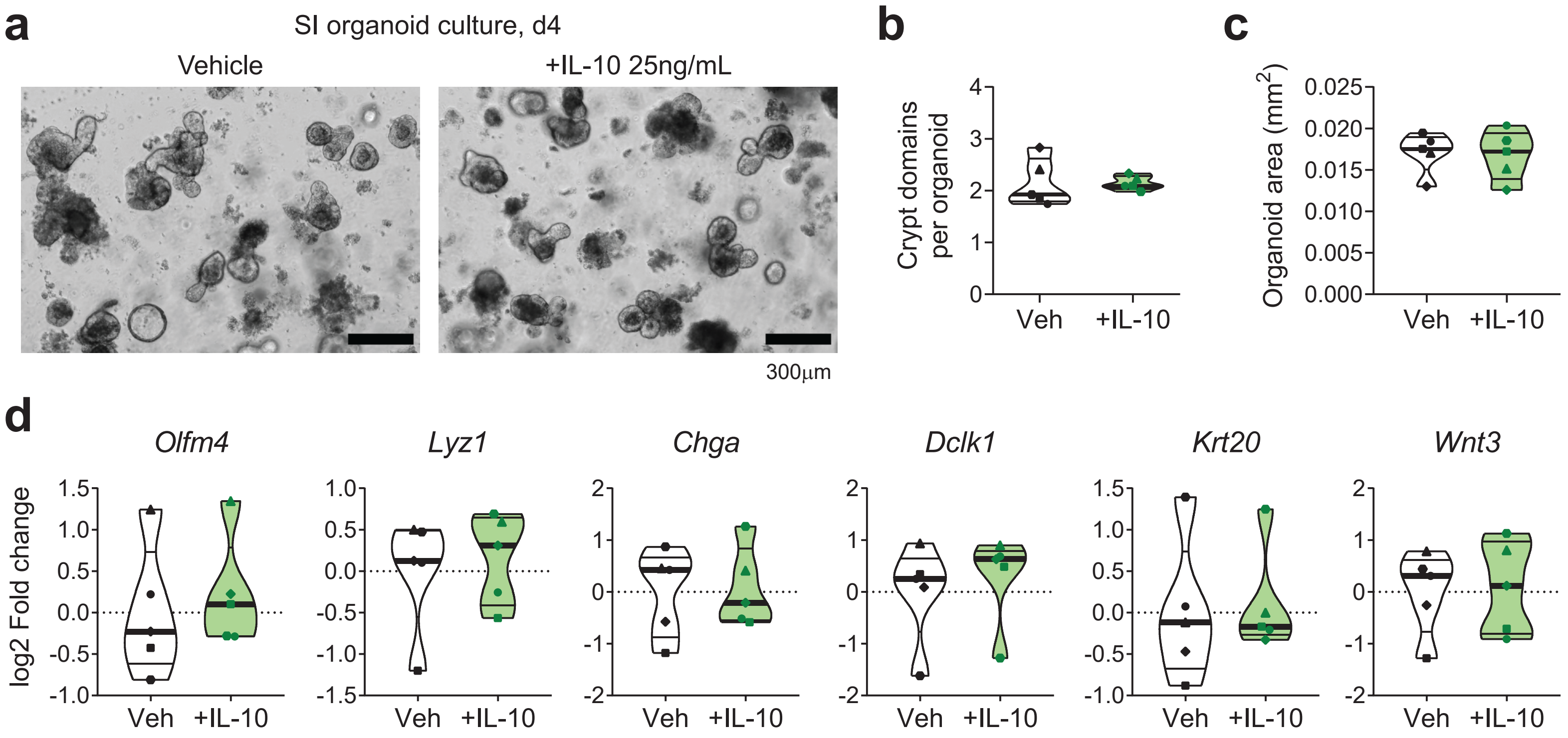

a
SI organoid culture, d4
Vehicle
+IL-10 25ng/mL
300μm
b
Crypt domains per organoid
Veh
+IL-10
c
Organoid area (mm²)
Veh
+IL-10
d
Olfm4
Lyz1
Chga
Dclk1
Krt20
Wnt3
log2 Fold change
Veh
+IL-10

Figure S8. Analysis from mouse SI organoids. (**a**) Bright-field images from the organoids grown after 4 days of treatment with 25ng/mL of recombinant murine IL-10. Scale bar = 300µm. (**b**) Quantification of the crypt domains per organoid and organoid areas in control and IL-10-treated organoids at d4 of culture. The average of each independent experiment is shown. (**c**) Transcriptomic analysis of markers for stem cells, secretory cells, and Wnt members by qRT-PCR. In **b** and **c**, each dot represents an independent organoid culture started from the crypts of 1 mouse. Paired two-way t-tests were performed in both **b** and **c**.

Figure S9

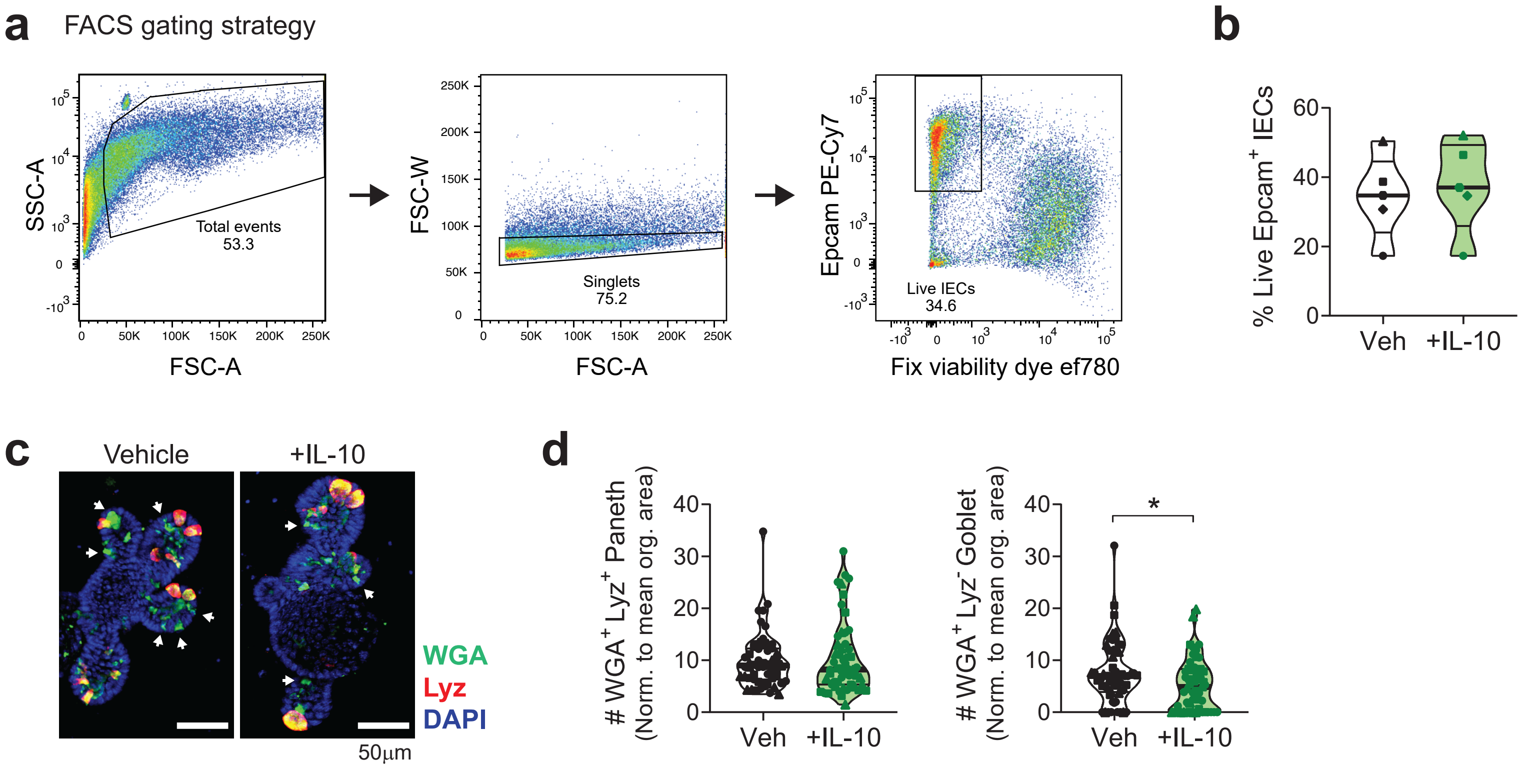

a
FACS gating strategy
SSC-A
FSC-A
Total events
53.3
FSC-W
Singlets
75.2
Epcam PE-Cy7
Fix viability dye ef780
Live IECs
34.6
b
% Live Epcam+ IECs
Veh
+IL-10
c
Vehicle
+IL-10
WGA
Lyz
DAPI
50μm
d
WGA+ Lyz+ Paneth
(Norm. to mean org. area)
WGA+ Lyz- Goblet
(Norm. to mean org. area)
*

Figure S9. FACS and immunofluorescence staining of SI organoids after IL-10 treatments. (**a**) Gating strategy for the selection of live Epcam^{+} intestinal epithelial cells (IECs). (**b**) Frequency of live Epcam^{+} IECs after treatments (1 dot = 1 independent mouse). (**c**) Whole-mount immunofluorescence pictures of d4 SI organoids stained with WGA and Lysozyme (Lyz) after IL-10 treatments. Goblet cells (WGA^{+} Lyz^{-}) are indicated with white arrows. (**d**) Quantification of Paneth (WGA^{+} Lyz^{+}) and goblet (WGA^{+} Lyz^{-}) cells per organoid. Dots represent measurements from individual organoids collected in 3 independent experiments. Scale bar = 50μm. A paired two-tailed t-test was performed in **b**, whereas unpaired two-tailed t-tests were used in **d**.

Table S1. List of oligos used for mutagenesis, cloning and DNA analysis.

a. Oligos used for CRISPR/Cas9 mutagenesis

Mutation	sgRNA sequence (5' - 3') (PAM in red)	Generated mutation
il10^{uu1751}	GGGCTTTCCTTTAAGACTGAGGG	-4bp (4bp deletion)
il10^{uu1762}	GGATTCTTCTGAAGAGCTGTTGG	+14bp insertion (3bp deletion,+17bp insertion)

b. Primers for genotyping

Mutation	Forward primer (5' - 3')	Reverse primer (5' - 3')
il10^{uu1751}	CTCTGGAGTCATCCTTTCTGCT	AGCTGGAGATCAGTCGAAGTGT
il10^{uu1762}	CCAACTTCAGTGACTAAAGGATTC (WT)<br>CATTCTGCCAACAGCTCTTCAG (KO2)	CCAAAACACTTACGCATTTCACC

c. Primers used for cloning of cDNA sequences

Gene	Forward primer (5' - 3')	Reverse primer (5' - 3')
il10	ATGATTTTCTCTGGAGTCATCCTTTCTGCAAAA	TTAGTGCTTAACCCTCTTTGAGGCTAGATACTGC
il10ra	AACTGCTTGGGTGCTACAGA	CCTTCACCGTATTTGGCCCT
il10rb	TCCAGTGTGACTCTGCGTTC	CCTCTGGGATGTGTGTGTCC
Il17a/f3	ATGCGGCTCTCACGGGTTTTC	CTAGTTCTGAGGCAAAACAATTGGTAAAACACATG

d. Primers used for microbiota analysis

Target	Forward primer (5' - 3')	Reverse primer (5' - 3')
Universal 16S bacterial rRNA	ATTACCGCGGCTGCTGGC	ACTCCTACGGGAGGCAGCAGT
Zebrafish Genomic (*il23r*)	AGACATACACTGAACGCTGTGG	TTTCTGTGTTGCAGAGCGTAAT

Table S2. List of primers used for qRT-PCR

Zebrafish

Gene	Forward primer (5' - 3')	Reverse primer (5' - 3')
eef1al1 (ef1a)	ACCTACCCTCCTCTTGGTCG	GGAACGGTGTGATTGAGGGAA
il1b	ATCAAACCCCAATCCACAGAGT	GGCACTGAAGACACCACGTT
tnfa	GGAGAGTTGCCTTTACCGCT	TTGCCCTGGGTCTTATGGAG
il17a/f3	CGCCTTGGACATACACAACTT	AGTAAATGGGTTGGGACTCCA
ifng1	GCTATGGGCGATCAAGGAAA	GCCGTCTCTTGCGTTCTTT
il22	CGATGACTGATACAGCACGA	TGTGCTCGTCTGATTCCAAG
cldn15la	TGTTTGATCGGCTGGGTTCT	AGCCTGAATGTGAACTGGCAA
fabp2	TGGGCGTCACCTTTGACTAT	GCGTGTCTCCCTCTATGACC
il10 (FR)	TCACGTCATGAACGAGATCC	CCTCTTGCATTTCACCATATCC
il10 (SE)	TAAAGCACTCCACAACCCCA	GACCCCCTTTTCCTTCATCTTTTC
il13	GAAGTGTGAGCATGATTATTTC	CTCGTCTTGGTGGTTGTAAG
il4	CATCCAGAGTGTGAATGGGA	TTCCAGTCCCGGTATATGCT
her6	AGAGCGAGAATCAACGAAAGC	TTTAGGGCAGCGGTCATTTG
her9	GTTCAGATGAGCGCAGCCTT	GCCAAATGAGCCTGTTGAGC
cyp26a1	GATGCTCTGGAGCACTACATTC	GTTCTTGCTCGTCCGTCTTTAT
atoh1a	TCCTGTCAAAGTACGCGAGC	CAATCCGTGCATTCTTCGCC
ascl1a	TGAGCGTTCGTAAAAGGAAACT	TGGCTCTTTGACACTCGGAC
olfm4.2	ACAGTCTCTACGCTGAGATTCAC	GCTTGGAGTCCTTTCCCCATC
sox9b	GCCCAGACGGAGGAAATCAG	TGAGACTGACCGGAGTGTTCT
muc2.1	TGCAACCTCAGCACATGGAT	TCCAGCTGTCGACAAACTCC
agr2	AGTGCTCTTGGTCATGGTGG	AGGGGCTTGTTCTTGGATCG

Mouse

Gene	Forward primer (5' - 3')	Reverse primer (5' - 3')
B2m	ACCGTCTACTGGGATCGAGA	TGCTATTTCTTTCTGCGTGCAT
Hes1	GAAAAATTCCTCCTCCCCGGT	GGCTTTGATGACTTTCTGTGCT
Atoh1	TCCCACAGAAGTGACGGAGA	GGGATATTTGTCACGGGGCT
Muc2	CAAGTGATTGTGTTTCAGGCTC	TGGAGATGTTCTTGGTGCAG
Rgcc	GTCACTCCTCGGAAAGCCAA	AGCTTCACTCTCCGAACTGC
Wnt3	TGGAACTGTACCACCATAGATGAC	ACACCAGCCGAGGCGATG
Chga	CAGCAGCTCGTCCACTCTTT	GACGCACTTCATCACCTTGG
Lyz1	CTGACTGGGTGTGTTTAGCTCAG	AATTGATCCCACAGGCATTCTT
Dclk1	CAGGAGTTTCTGTAATAGCAACCA	CCGAGTTCAATTCCGGTGGA
Olfm4	TGCTCCTGGAAGCTGTAGTCA	TGTATTCAAAGGTGCCACCCA
Krt20	GTCCCACCTCAGCATGAAAGA	TCTGGCGTTCTGTGTCACTC
